# Comparative Proteomic and Metabonomic Profiling of Buds with Different Flowering Capabilities Reveal Novel Regulatory Mechanisms of Flowering in Apple

**DOI:** 10.3390/plants12233959

**Published:** 2023-11-24

**Authors:** Shujin Wang, Xiaoping Chen, Sitong Liu, Xiaochen Zhang, Yu Li, Wei Shang, Jiahui Song, Jianwen Tian, Xiaolong Li, Libo Xing

**Affiliations:** 1 College of Horticulture, Northwest A & F University, Xianyang 712100, China; wangshujin@nwafu.edu.cn (S.W.); chenxp@nwafu.edu.cn (X.C.); 2022050305@nwafu.edu.cn (S.L.); xiaochen_zhang@nwafu.edu.cn (X.Z.); l_i_yu@nwafu.edu.cn (Y.L.); sxtysw626@nwafu.edu.cn (W.S.); sjjhhh@nwafu.edu.cn (J.S.); 2 Ningxia Academy of Agriculture and Forestry Science, Institute of Horticulture, Yinchuan 750002, China; tjw6789@126.com (J.T.); xiaolong85115@163.com (X.L.)

**Keywords:** apple, flowering, proteomic, metabolomic, carbohydrate, amino acid, organic acids

## Abstract

Flower bud formation in the apple tree life cycle is associated with multiple biological processes. To explore the physiological and molecular mechanisms underlying the protein and metabolite changes in buds with different flowering capabilities, axillary buds with no flowering (Ab), long-shoot buds with a low flowering rate (Lb), and spur buds with a higher flowering rate than the Lb (Sb) were analyzed using a Tandem Mass Tag™ proteomic technique in combination with nLC–MS/MS analyses. We identified 471 (88 up- and 383 down-regulated), 459 (176 up- and 283 down-regulated), and 548 (387 up- and 161 down-regulated) differentially expressed proteins in Sb vs. Lb, Sb vs. Ab, and Lb vs. Ab, respectively, that were involved in carbohydrate, amino acid and lipid transport, and metabolism. Additionally, 110 (91 increased and 19 decreased), 89 (71 increased and 18 decreased), and 99 (37 increased and 62 decreased) metabolites having significantly different levels were identified in Sb vs. Lb, Sb vs. Ab, and Lb vs. Ab, respectively. The identified metabolites were related to amino acids and their isoforms, sugars and polyols, and organic acids, and occurred at significantly greater levels in the Sbs than the other buds. Thus, flower bud formation is a complex process that involves various biochemical materials and signals, such as carbohydrates, amino acids and their isoforms, and organic acids.

## 1. Introduction

In 2021, 45.98 million tons of apple (*Malus domestica* Borkh.) were produced in China, accounting for 49% of world production [1]. ‘Fuji’, as the main cultivar variety, with a long storage capacity, firmness, and good quality, was grown on 70% of the total cultivation area for apples in China, but they have serious production problems, including a long ripening period, poor quality flower buds, and alternate bearing [2,3,4,5], which seriously affect their yield and economic benefits. Thus, a better understanding of the factors influencing flower bud formation and its physiological molecular regulatory mechanisms could help to address the flowering-related problems in apple trees.

Floral induction and flower bud formation in woody plants are ongoing processes that occur in separate growing seasons [2,6], and complex regulatory networks are involved in multiple floral pathways (i.e., autonomous, thermosensitivity, photoperiod, sugar, and aging) [7,8,9,10] which are affected by multiple environmental and internal signals that ensure the appropriate timing of flowering [6,8,11]. Multiple biochemical materials and signals, such as carbohydrates, polyamines [12,13,14], fatty acids, and lipids [15], as well as secondary metabolites [15,16], play important roles in regulating floral induction and flower bud formation.

A “sugar signal pathway” in flowering plants that induces flower bud formation by regulating carbohydrate metabolism has been elucidated [2,6]. Carbohydrates, as signaling and energy substances, such as sucrose, glucose, and trehalose-6-phosphate, have key roles in multiple flowering-related pathways that lead to floral induction and flower bud formation [6,17,18,19]. Among them, signals from trehalose-6-phosphate, the central regulator of the ‘C’ pathway, are usually produced by the meristem in response to the environmental and carbohydrate status and determine whether plants start to flower [6]. Metabolic substances, such as fatty acids and phosphatidylcholine (PC), which are involved in carbon and lipid metabolic pathways, also play key roles in the regulation of floral induction and flower bud formation in plants [4,15]. 

Amino acids, as necessary substrates for protein biosynthesis in plant life cycles, are the key ‘material basis’ and ‘signal molecules’ involved in multiple biological processes, such as flowering, programmed cell death, and growth [12,20,21,22]. For example, proline, as a signal molecule, can actively participate in the regulation of plant flowering [20], and spraying proline significantly promotes Vigna early flowering [22]. The overexpression of enzyme-encoding genes important for proline synthesis, the Vigna *P5SC* gene in tobacco [23] and *AtP5CS1* in *Arabidopsis thaliana* [20], results in the early flowering phenotype. Additionally, polyamines play key roles in cell division and differentiation, embryonic development, fruit setting, flowering, and dormancy [13,14]. The levels of these polyamines are different in floral and vegetative buds, with the former containing greater amounts of conjugated polyamines [24]. For example, relatively greater levels of polyamines during flower development may contribute to the differentiation and maturation processes of azalea flowers [25].

To increase the understanding of the molecular regulatory mechanisms and different levels of proteins and metabolites during floral induction in apple buds with different flowering capabilities, we identified and investigated whether protein and metabolic substance levels, which may contribute to flower bud formation, changed among buds having different flowering capabilities. The buds used in the study were axillary buds with no flowering (Ab), long-shoot buds with a low flowering rate (Lb), and spur buds with a higher flowering rate than the Lb (Sb). Tandem Mass Tag™ (TMT) and nLC–MS/MS analyses were used as effective methods to compare the proteomic and metabolomic profiles of buds with different flowering capabilities. The results provide a better understanding of the molecular regulatory mechanisms of floral induction and flower bud formation in apple trees. 

## 2. Results

### 2.1. The Phenotypes of Buds with Different Flowering Capabilities and Their Flowering Rates 

**Figure 1:**
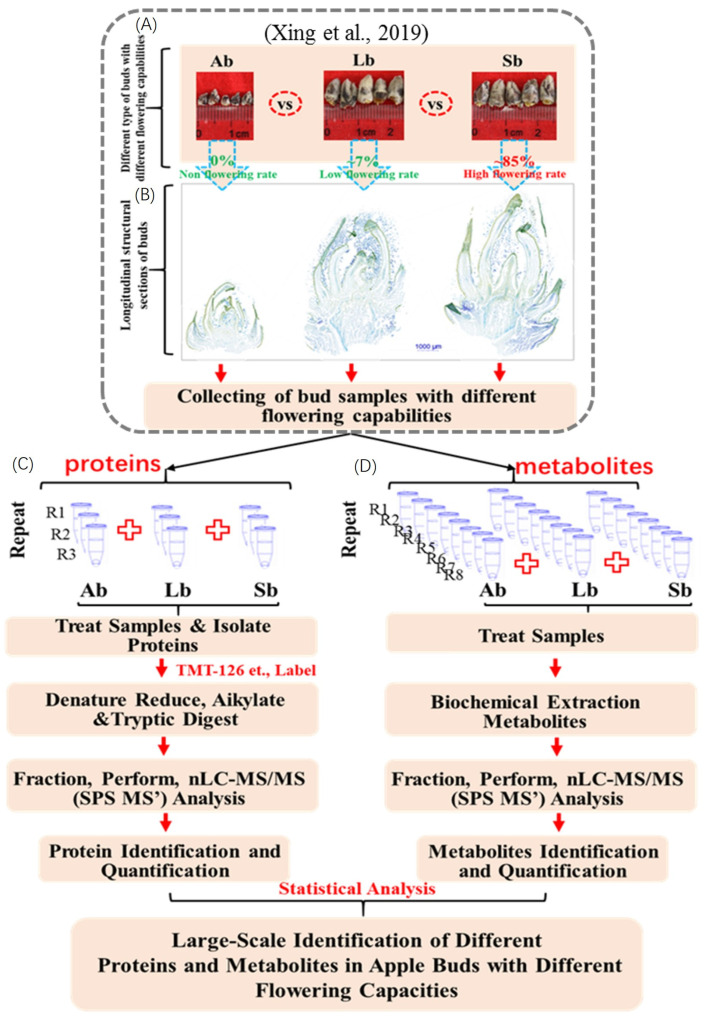
Experimental design for the proteomic and metabolomic analyses of apple buds with different flowering capabilities using the Tandem Mass Tag™ and nLC–MS/MS methods. (**A**) The phenotypes of buds. (**B**) The longitudinal structural sections of buds. (**C**,**D**) The flow of proteomic and metabolomic analyses of buds. Ab: axillary buds with no flowering; Lb: long-shoot buds with a low flowering rate; and Sb: spur buds with a higher flowering rate. The bud samples with different flowering capabilities for proteins and metabolites were the same as in our previous publication [26].

**Figure 2:**
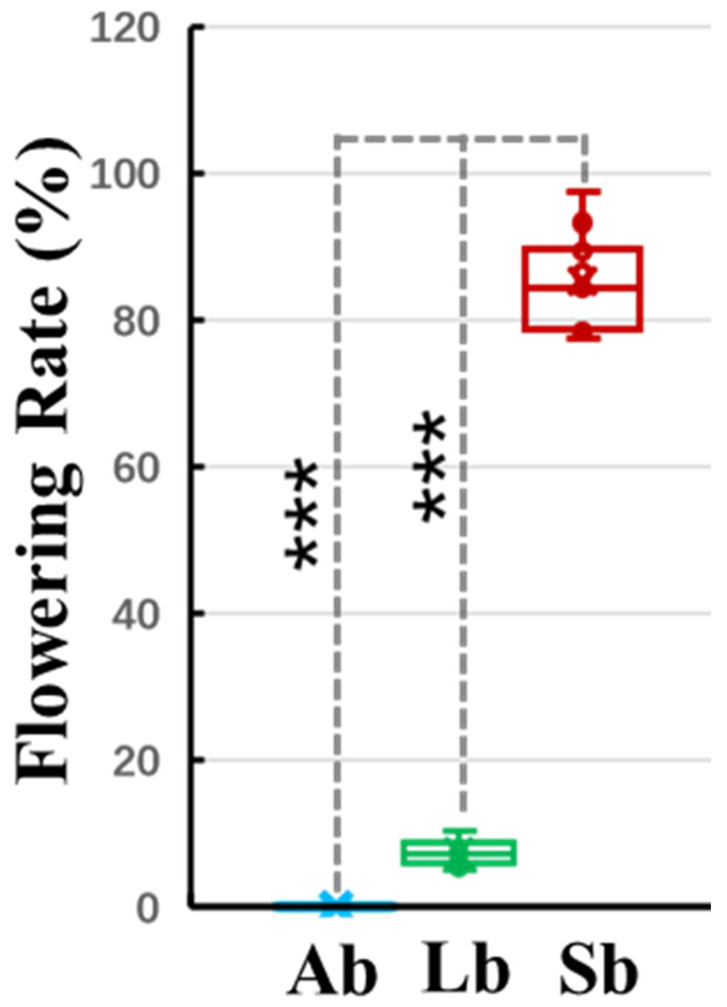
Flowering rates of apple buds with different flowering capabilities. Ab: axillary buds with no flowering; Lb: long-shoot buds with a low flowering rate; and Sb: spur buds with a higher flowering rate than the Lb. Data represent the means ± SEs, *n* = 10; ***, *p* < 0.001; and ns, non-significant (*p* > 0.05).

We collected bud samples with different flowering capabilities from different axillary, long, and spur shoots in ten-year-old ‘Nagafu No. 2’, a cultivar of ‘Fuji’ apple (*M. domestica* Borkh.) trees. The sizes and longitudinal structural sections of apple buds with different flower capabilities are shown in Figure 1. The lengths and widths of the Sbs were significantly greater than those of the Abs and Lbs (Figure 1). The flowering rates of Sbs, at ~85%, were significantly greater than those of Abs and Lbs, ~0% and ~7%, respectively (Figure 1 and Figure 2). In order to analyze the samples by Tandem Mass Tag TM and nLC–MS/MS, we extracted and labeled the total proteins of different sample bud samples with different flowering capabilities (Ab, Lb, and Sb). Protein extractions were performed in three biological replicates, with each biological replicate consisting of an independent pool of nine bud samples.

### 2.2. Primary Data Analysis, and Protein and Metabolite Identification in Buds with Different Flowering Capabilities

We identified the proteins and metabolites in apple buds with flowering capabilities using the TMT technique and GC-MS, respectively; the detailed experimental design for the proteomic and metabolomic analyses is shown in Figure 1. The isoelectric point distribution of identified proteins is shown in Figure S1A, with isoelectric points of 6, 7, and 9 being the top three among the identified proteins. The distribution of the peptide numbers is shown in Figure S1B, with peptide counts of 0–1, 1–2, and 2–3 being the top three counts of identified proteins. The molecular weight distribution of the identified proteins is shown in Figure S1C, with molecular masses of 20–30, 30–40, and 40–50 kDa being the top three mass ranges of identified proteins. Additionally, a protein sequence coverage of 0–5%, 5–10%, and 10–15% were the top three ranges of identified proteins (Figure S1D). The correlations among sequencing protein data in each apple bud sample, with three biological replicates, are shown in Figure S2. In total, 6898 quantified proteins were identified in buds with different flowering capabilities, and their detailed information is shown in Additional file S1. 

To precisely measure the metabolic changes in apple buds with different flowering capabilities, the metabolites of each type of bud, with eight biological replicates, were measured by GC-MS (Figure 1). In total, 230 metabolites were measured and identified. These were mainly involved with amino acids and their isoforms, amines, organic acids, fatty acids, sugars, and polyols (Additional file S2). 

### 2.3. Venn Diagrams and Heatmap Analyses of DEPS in Buds with Different Flowering Capabilities

**Figure 3:**
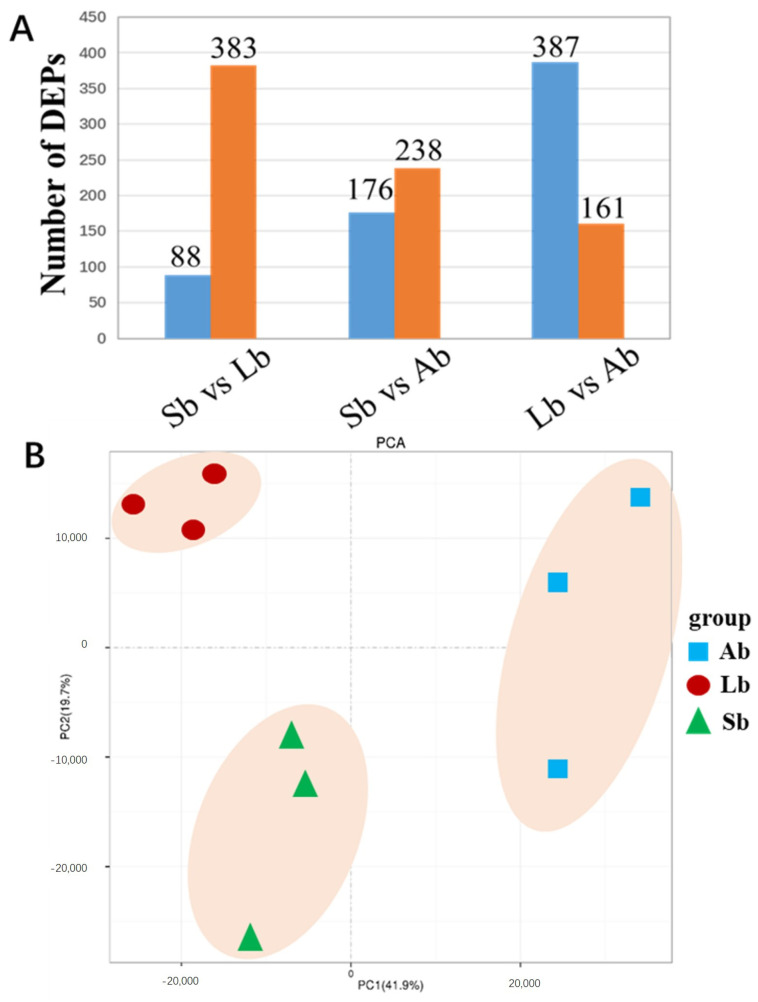
The number of differentially expressed proteins (DEPs) and PCA score plots for apple buds with different flowering capabilities. Ab: axillary buds with no flowering; Lb: long-shoot buds with a low flowering rate; and Sb: spur buds with a higher flowering rate than the Lb. (**A**) The number of differentially expressed proteins (DEPs) for apple buds with different flowering capabilities. (**B**) The PCA score plots for apple buds with different flowering capabilities.

The number of DEPs that were significantly different (log 2 fold change > 2; FDR < 0.05) among buds with different flowering capabilities was investigated (Figure 2 and Figure 3). We found 471 (88 up- and 383 down-regulated), 459 (176 up- and 283 down-regulated), and 548 (387 up- and 161 down-regulated) DEPs in Sb vs. Lb, Sb vs. Ab, and Lb vs. Ab, respectively (Figure 3A). PCA plots were generated for each bud sample and a clear separation among Abs, Lbs, and Sbs was found, indicating that clear protein differences existed among these different types of buds (Figure 3B). 

**Figure 4:**
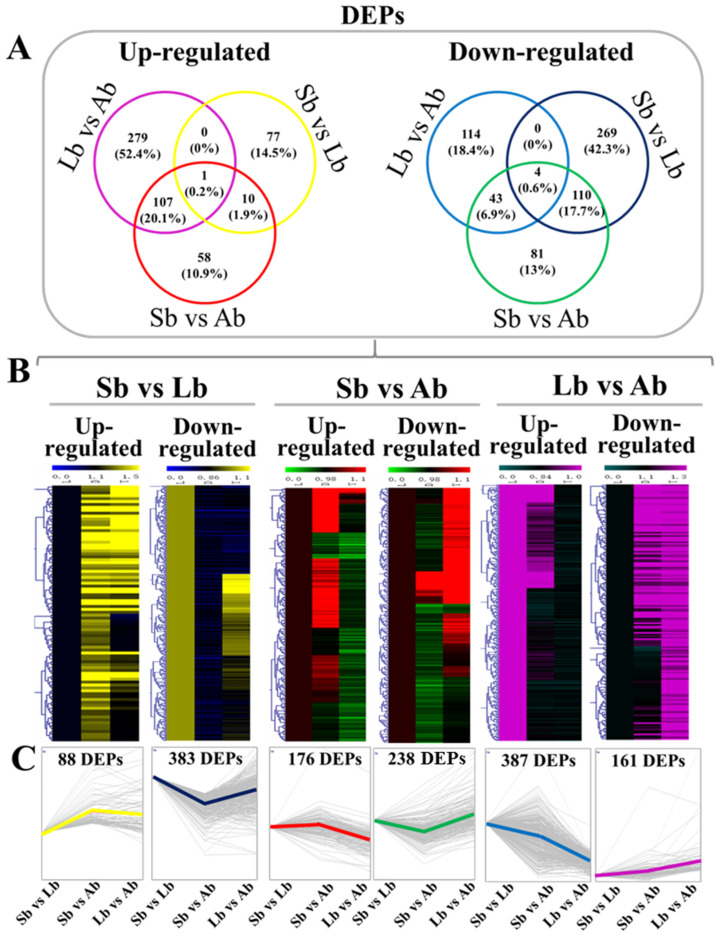
Venn diagram and heatmap analysis of the differentially expressed proteins (DEPs) in apple buds with different flowering capabilities. (**A**) Venn diagrams showing unique and common DEPs, including up-regulated and down-regulated proteins, in the three different comparison groups; and (**B**,**C**) heatmap and cluster analyses of DEPs in different comparison groups. Ab: axillary buds with no flowering; Lb: long-shoot buds with a low flowering rate; and Sb: spur buds with a higher flowering rate than the Lb.

Additionally, Venn diagrams showed unique and common DEPs, including those of up- and down-regulated genes, in the three different comparison groups (Sb vs. Lb, Sb vs. Ab, and Lb vs. Ab; Figure 4A), and heatmap and cluster analyses of DEPs in different comparison groups are shown in Figure 4B,C, which indicated that there were significant differences in the identified proteins of buds with different flowering capabilities.

### 2.4. Functional Categories of DEPS in Buds with Different Flowering Capabilities

**Figure 5:**
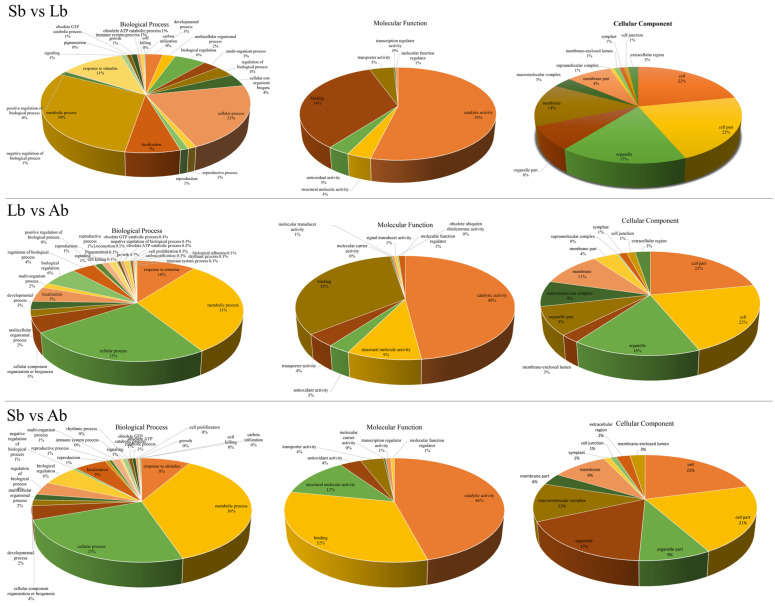
The number of differentially expressed proteins in apple buds with different flowering capabilities involved in molecular functions and cellular components during floral induction. Ab: axillary buds with no flowering; Lb: long-shoot buds with a low flowering rate; and Sb: spur buds with a higher flowering rate than the Lb.

We performed a GO functional analysis of the DEPs among the buds in different comparison groups (Sb vs. Lb, Sb vs. Ab, and Lb vs. Ab) to identify the enriched biological processes, cellular components, and molecular functions (Figure 5). The most highly enriched biological processes in the Sb vs. Lb group were the metabolic process proteins (30%), followed by cellular process proteins (21%) and response to stimulus proteins (11%) (Figure 5); similar results were found for the Sb vs. Ab and Lb vs. Ab groups (Figure 5). For molecular functions, the most highly enriched category was catalytic activity proteins (54%, 46%, and 48% for the Sb vs. Lb, Sb vs. Ab, and Lb vs. Ab groups, respectively), followed by binding proteins (34%, 32%, and 33% for the Sb vs. Lb, Sb vs. Ab, and Lb vs. Ab groups, respectively) (Figure 5). The most highly enriched cellular component was cell proteins (22%, 21%, and 22% for the Sb vs. Lb, Sb vs. Ab, and Lb vs. Ab groups, respectively), followed by cell part proteins (22%, 21%, and 22% for the Sb vs. Lb, Sb vs. Ab, and Lb vs. Ab groups, respectively) (Figure 5). 

The KEGG analysis of the DEPs in the different comparison groups (Sb vs. Lb, Sb vs. Ab, and Lb vs. Ab, respectively) is shown in Figure S3. The KEGG pathways containing the most DEPs for the Sb vs. Lb group were protein processing in the endoplasmic reticulum (20 proteins), phenylpropanoid biosynthesis (14 proteins), and flavonoid biosynthesis (11 proteins) (Figure S3). For the Sb vs. Ab group, the most highly enriched pathways were ribosome (53 proteins), phenylpropanoid biosynthesis (13 proteins), and flavonoid biosynthesis (13 proteins) (Figure 5). The most highly enriched pathways in the Lb vs. Ab group were ribosome (41 proteins), protein processing in the endoplasmic reticulum (20 proteins), and starch and sucrose metabolism (14 proteins) (Figure 5).

### 2.5. DEPs Related to Carbohydrate, Amino Acid, and Lipid Transport and Metabolism 

The data showed numerous changes in the expression levels of the proteins involved in carbohydrate synthesis, metabolism and transport processes, amino acid transport and metabolism, and the fatty acid and lipid pathways (Table 1). For example, probable fructose-bisphosphate aldolase 2 (FBA2: MD10G1063600), granule-bound starch synthase 1 (GBSSIb:MD07G1159300), probable beta-D-xylosidase 5 (BXL5: MD05G1099400/MD07G1210800), and ribulose bisphosphate carboxylase large chain (RBCL: MD02G1049600) proteins associated with carbohydrate metabolism were identified as being up-regulated in Sbs compared with Lbs (Table 1). Similarly, hexokinase 2 (HXK2: MD09G1138500), peroxidase (PAP26: MD02G1021200), and sucrose synthase 7 (SUS7: MD17G1287000) proteins were also significantly higher in Sbs compared with Abs, as well as in Lbs compared with Abs (Table 1). Additionally, hexokinase 1 (HXK1: MD09G1088100), beta-galactosidase 9 (BGAL9: MD13G1283100), and probable sucrose-phosphate synthase 1 (SPS1: MD09G1214300) proteins were significantly higher in Lbs compared with Abs (Table 1). 

Additionally, two (R)-mandelonitrile lyases (MDL2: MD00G1013000 and MDL3: MD03G1091100), phospho-2-dehydro-3-deoxyheptonate aldolase 1 (DHAPS1: MD00G1037100), serine carboxypeptidase-like 50 (SCPL50: MD16G1027500), aspartate aminotransferase (ASP1: MD17G1264900), and phenylalanine ammonia-lyase 1 (PAL1: MD04G1096200) proteins involved in amino acid transport and metabolism were significantly lower in Sbs, which have the highest flowering rate compared with Lbs and Abs (Table 1). However, argininosuccinate synthase (At4g24830: MD08G1219100), chorismate synthase (EMB1144: MD08G1183900), glutamate dehydrogenase 1 (GDH1: MD08G1207000) and methylenetetrahydrofolate reductase 2 (MTHFR2: MD08G1184000) proteins were significantly higher in Lbs compared with Abs (Table 1). 

In lipid pathways, proteins, including 1,4-dihydroxy-2-naphthoyl-CoA synthase (MENB: MD13G1208400), 3-hydroxyisobutyryl-CoA hydrolase 1 (CHY1: MD02G1229900), 4-coumarate-CoA ligase 1 (4CL1: MD17G1229400), acetyl-coenzyme A carboxylase carboxyl transferase subunit alpha (CAC3: MD08G1051100), and sphinganine C (4)-monooxygenase 2 (SBH2: MD16G1073700), which are involved in lipid transport and metabolism, were significantly lower in Sbs compared with Lbs, but showed significantly greater levels in Lbs compared with Abs (Table 1). In addition, five Hsp20/alpha crystallin family proteins (MD01G1144400; MD07G1210700; MD11G1087100; MD01G1208700; and MD07G1279100) had significantly greater expression levels in Sbs compared with Lbs (Table S1). However, seven cytochrome P450 family proteins, including CYP98A2: MD15G1436500; CYP82C4: MD05G1170000; CYP82A4: MD15G1028400/MD08G1234700; and CYP82A3: MD15G1028200, had significantly lower expression levels in Sbs compared with Lbs and Abs (Table S1). 

### 2.6. Metabolic Profiles of Buds with Different Flowering Capabilities

**Figure 6:**
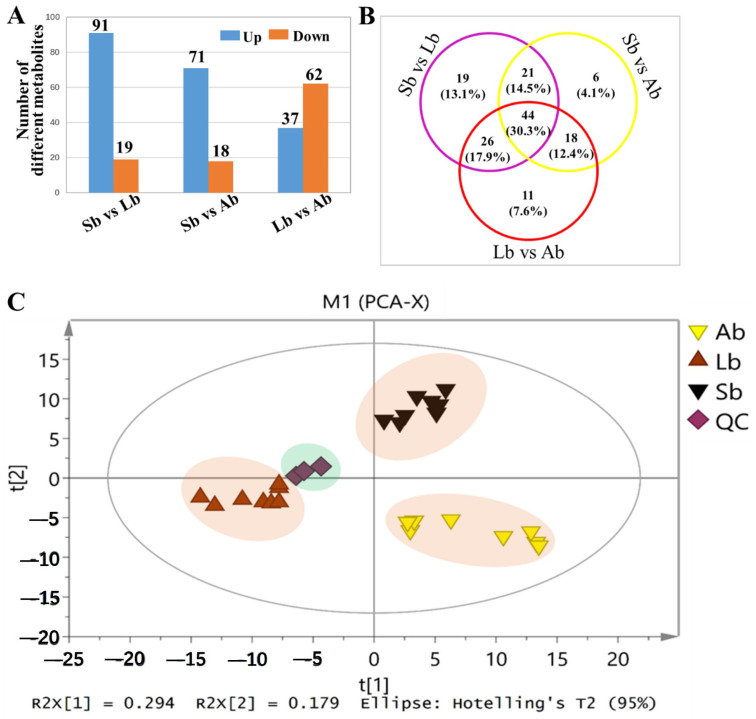
Analysis of identified metabolites present at varying levels in apple buds with different flowering capabilities. (**A**) The number of different metabolites in buds. (**B**) Venn diagrams showing unique and common metabolites, including up-regulated and down-regulated proteins, in the three different comparison groups. (**C**) PCA. The score plots for buds with different flowering capabilities showing clear metabolic differences among the samples. Ab: axillary buds with no flowering; Lb: long-shoot buds with a low flowering rate; and Sb: spur buds with a higher flowering rate than the Lb.

The metabolic profiles of buds with different flowering capabilities were investigated (Figure 6; Table 2 and Table S2). We identified 110 (91 increased and 19 decreased), 89 (71 increased and 18 decreased), and 99 (37 increased and 62 decreased) metabolites that showed significantly different levels in Sb vs. Lb, Sb vs. Ab, and Lb vs. Ab, respectively (Figure 6A). Venn diagrams showed the unique and common metabolites, including those with increased and decreased levels, in the three different comparison groups (Sb vs. Lb, Sb vs. Ab, and Lb vs. Ab) (Figure 6B). Additionally, bud samples with different flowering capabilities (including eight biological replicates) clustered in the PCA mainly based on the flowering rate of the buds. For example, eight buds with ~85% flowering rates (Sbs) were clustered tightly, indicating clear metabolic differences among the bud samples with different flowering capabilities (Figure 6C and Figure S4D–F). In addition, other samples of different types of buds with different flowering rates were divided into different clusters based on their flowering capabilities (Figure 6C and Figure S4D–F).

The results indicated that there were significant differences among the metabolite levels of different types of buds. A supervised PLS-DA was performed to investigate the metabolic variations among buds with different flowering capabilities (Figure S4G–I). The PLS-DA score plots showed that the metabolic profiles were clearly distinguishable based on the flowering rates of the buds (Figure S4G–I). The model parameter R^2^Y was used to evaluate the quality of the PLS-DA model. The models had high R^2^Y values of 0.861, 0.822, and 0.766 for Sb vs. Lb, Sb vs. Ab, and Lb vs. Ab, respectively, which indicated that the models had high explanatory and satisfactory predictive capabilities (Figure S5). 

### 2.7. Clustering and Heatmaps of Metabolites in Buds with Different Flowering Capabilities 

**Figure 7:**
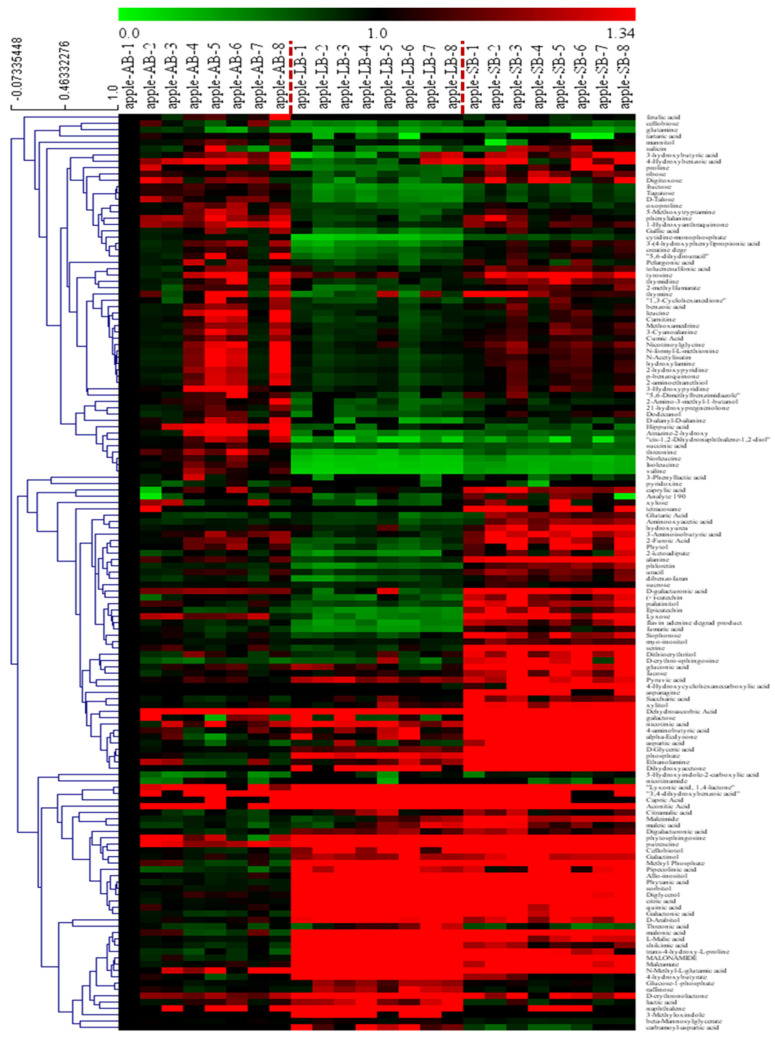
Clustered heatmap of the normalized metabolites in apple buds with different flowering capabilities. Ab: axillary buds with no flowering; Lb: long-shoot buds with a low flowering rate; and Sb: spur buds with a higher flowering rate than the Lb. Each bud sample had eight biological replications.

Heatmap and cluster analyses of the metabolites in buds with different flowering capabilities are shown in Figure 7 and indicated that there were significant differences among the identified metabolites in buds with different flowering capabilities. The metabolic profiles of these buds were analyzed using hierarchical clustering, which grouped these metabolites into three major clusters mainly based on amino acids and their isoforms, sugars and polyols, and organic acids (Figure 7; Table 2 and Table S2; Additional file 2). Approximately 24 amino acids and their isoforms (such as alanine, proline, threonine, and phenylalanine), 25 sugars and polyols (such as sucrose, fructose, tagatose, and sophorose), organic acids (such as pyruvic, pipecolinic, aconitic, and galactonic acids) and others (such as phosphate, thymidine, and phloretin) were identified and showed significant differences in their levels among buds with different flowering capabilities (Table 2 and Table S2). 

**Figure 8:**
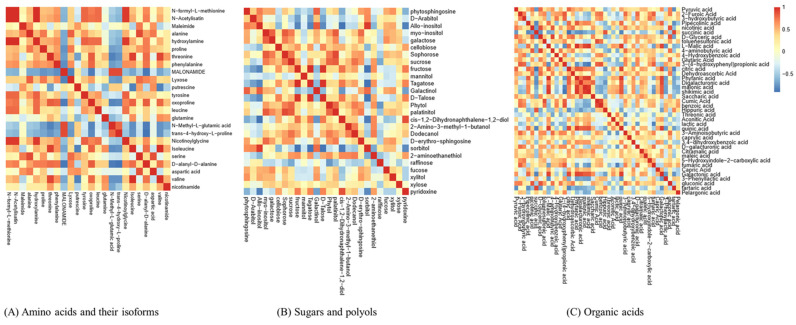
A heatmap was used to represent the significant statistical correlation values (r) among the metabolites in apple buds with different flowering capabilities. Ab: axillary buds with no flowering; Lb: long-shoot buds with a low flowering rate; and Sb: spur buds with a higher flowering rate than the Lb. Blue squares indicate significant positive correlations (r > 0.5, *p* < 0.05), white squares indicate nonsignificant correlations (*p* > 0.05), and red squares indicate significant negative correlations (r < −0.5, *p* < 0.05).

In addition, a Pearson’s correlation analysis identified potential links among these metabolites in the bud samples (r > 0.5 or r < −0.5, *p* < 0.05) (Figure 8). Our correlation analysis identified multiple significant associations among the metabolites in buds with different flowering capabilities, as shown in Figure 8A–C.

### 2.8. Important Metabolic Pathways Associated with the Citrate (TCA) Cycle and Amino Acid Metabolism

**Figure 9:**
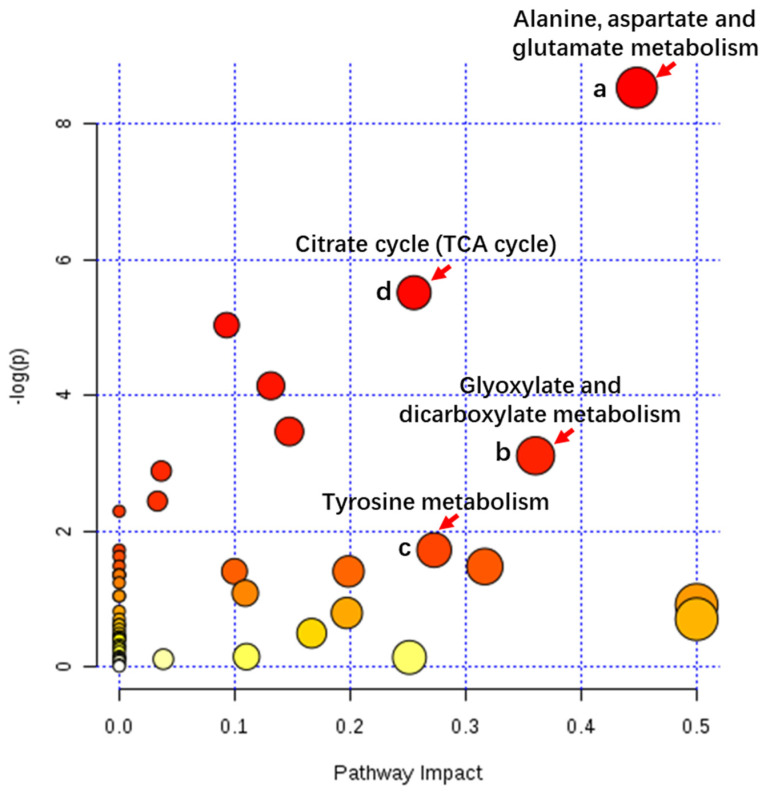
Summary of MetPA. (a) Glycine, serine, and threonine metabolism; (b) pantothenate and CoA biosynthesis; (c) nicotinate and nicotinamide metabolism; and (d) bile acid metabolism. Different colors indicate different levels of metabolites.

The impact value of a MetPA (pathway topology analysis) was used to evaluate the importance of the metabolite-related pathways in buds with different flowering capabilities (Figure 9, Table S3). As a result, eight metabolic pathways, including alanine, aspartate and glutamate metabolism, the TCA cycle, aminoacyl-tRNA biosynthesis, galactose metabolism, and arginine and proline metabolism (impact > 0.01), that are associated with amino acids and their isoforms, sugars and polyols, and organic acids were considered the most relevant (Figure 9; Table S3). Among them, three metabolic pathways, amino acid metabolism, sugar and polyol metabolism, and lipid and organic acid metabolism, were identified both by a statistical correlation analysis and by MetPA (Figure 8 and Figure 9). These identified metabolites belonging to KEGG pathways in buds with different flowering capabilities can be seen in Table S4. Thus, these pathways may play key roles in the formation of flower buds.

### 2.9. Metabolites Related to Amino Acids and Their Isoforms, Sugars and Polyols, and Organic Acids

**Figure 10:**
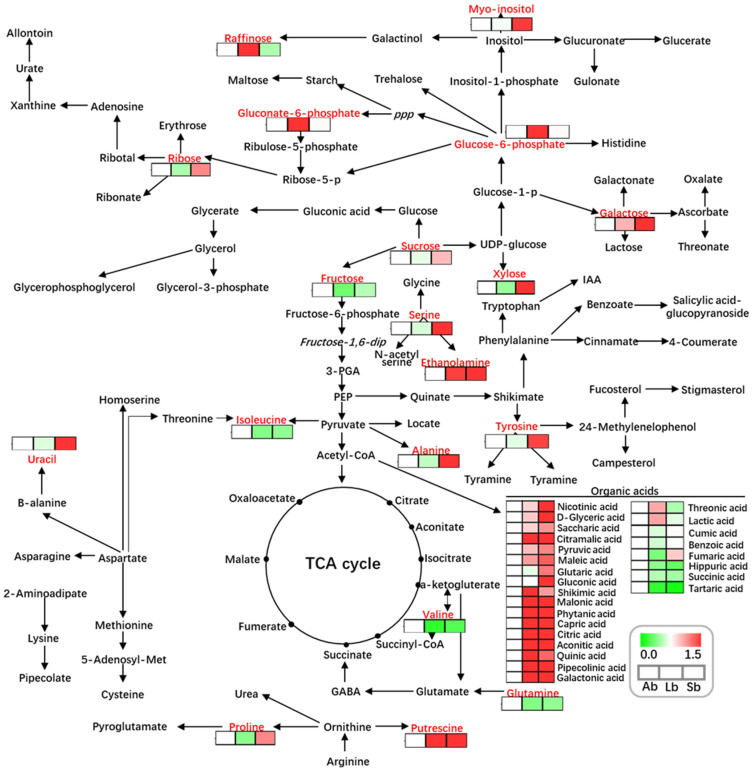
Model showing the metabolite changes involved in the TCA, sugar and amino acid, and polyamine cycles in apple buds with different flowering capabilities. Ab: axillary buds with no flowering; Lb: long-shoot buds with a low flowering rate; and Sb: spur buds with a higher flowering rate than the Lb.

The metabolic networks of the metabolites involved in nitrogen metabolism (such as amino acid metabolism, amine metabolism, and polypeptide metabolism), carbon metabolism (such as polysaccharide, monosaccharide, sugar, and polyol metabolism, and the TCA cycle), and organic acid metabolism were constructed based on the identified metabolites in buds with different flowering capabilities (Table 2 and Table S2; Figure 10). For example, approximately 18 amino acids and their isoforms (such as alanine, proline, threonine, phenylalanine, hydroxylamine, and N-acetylisatin) were present at significantly greater levels in Sbs, which have an ~85% flowering rate, than in Lbs, but were present at significantly lower levels in Lbs compared with Abs (Table 2). Other metabolites, including malonamide, trans-4-hydroxy-L-proline, and malonamide, were present at significantly lower levels in Sbs than in Lbs (Table 2). Similarly, ~18 sugars and polyols (such as sucrose, fructose, tagatose, fucose, xylose, sophorose, dodecanol, and 2-aminoethanethiol) were present at significantly greater levels in Sbs, which have the highest flowering rate, than in Lbs (Table 2), indicating that sugars and polyols may contribute to apple flower bud formation. Additionally, ~36% of the identified organic acids (such as pyruvic, pipecolinic, aconitic, galactonic, quinic, and citramalic acids) were present at significantly greater levels in Sbs than in Abs, which did not flower (Table 2 and Table S2). However, several organic acids (including succinic, hippuric, tartaric, and threonic acids) showed the opposite trend (Table 2 and Table S2). In addition, these important metabolites, including gluconate-6-phosphate, sucrose, myo-insitol, and fructose, which belong to carbon metabolism pathways, and isoleucine, serine, uracil, ethanolamine, alanine, tyrosine, valine, proline, putrescine, and glutamine, which are involved in nitrogen metabolism and the TCA cycle pathways, were present at significantly different levels among buds with different flowering capabilities (Figure 10). Thus, a pathway map of the differential metabolites in buds with different flowering capabilities involved in carbon, nitrogen, and organic acid metabolism (Figure 10) showed that the amino acids and their isoforms, sugars and polyols, and organic acids play key roles in bud growth and flower bud formation. 

## 3. Discussion

Floral induction and flower bud formation involve complex regulatory networks and are affected by internal signals (such as those from genes, DNA, RNA, proteins, and metabolites) [7,27,28,29], as well as multiple environmental factors, including age, light, temperature, and water [7,10,11], that ensure the appropriate timing of flowering. There are five key flowering pathways, including aging, photoperiod, gibberellic acid, sugar signaling, and thermosensory, which together determine the formation of flower buds in plants [20,30,31]. Rapid and comprehensive advancements in proteomics and metabolomic sequencing techniques have been widely used to study various biological functions and processes in plants, including embryo development, seed germination, transition phases, and flowering. ‘Fuji’ apples, which have limited inferior flower buds and biennial fruiting, are grown in 70% of the total cultivated apple area in China [2,3,32]. Here, we investigated and compared the dynamic changes in the proteins and metabolites in apple (*M. domestica* Borkh.) buds with different flowering capabilities, including Abs, Lbs, and Sbs, which have ~0%, 7%, and 85% flowering rates, respectively (Figure 1 and Figure 2), to gain insights into the regulatory mechanism of apple flower bud formation. 

The metabolism of carbohydrates, such as sucrose [6,17,19], glucose [33], and starch [34], as well as sugars and polyol metabolites [2,16,35], which are important energy and signaling substances, play key roles in flower bud formation across multiple flowering pathways [6,36]. The global dynamic changes in the proteins and metabolites in apple (*M. domestica* Borkh.) buds with different flowering capabilities indicated that the majority of identified DEPs and differential metabolites belonged to carbon metabolism, including carbohydrates, polysaccharides, monosaccharides, sugars, and polyols, the TCA cycle, amino acids (i.e., alanine, proline, threonine, and phenylalanine), lipid transport and metabolism, and organic acid pathways (Table 1, Table 2, Tables S1 and S2), indicating that these metabolites and their associated complex regulatory networks contribute to flower bud formation in apples. 

In our data, the sucrose level was significantly greater in Sbs, which had the highest flowering rate, than in Lbs, which had a ~7% flowering rate (Figure 1 and Figure 2). Proteins involved in sucrose biosynthesis, such as sucrose synthase 7 (SUS7: MD17G1287000) proteins, were up-regulated in Sbs compared with Abs, and probable sucrose-phosphate synthase 1 (SPS1: MD09G1214300) proteins were up-regulated in Lbs compared with Abs (Table 1). Spraying sucrose can significantly increase the flower buds of ‘Fuji’ apple trees, as well as the expression of flowering genes [5], and only 1% (*w*/*v*) sucrose can induce flower bud formation by activating the genes that control the floral transition [37]. The up-regulated genes involved in sucrose synthase (i.e., *SUS1* and *SUS4*) in the sugar and photoperiodic flowering pathways contribute to floral induction and increase the number of lower buds [19], suggesting that there is a positive correlation between sucrose levels and flower bud formation which involves the sugar signaling-mediated flowering pathway [17,19]. 

Additionally, in the whole carbon metabolic regulatory network, multiple sugar-related metabolites (such as sucrose, fructose, fucose, and gluconate-6-phosphate), as well as their synthesis- and metabolism-related proteins, such as probable fructose-bisphosphate aldolase 2 (FBA2: MD10G1063600) and hexokinase 2 (HXK2: MD09G1138500), were expressed at significantly different levels among buds with different flowering capabilities (Table 1), suggesting that a series of metabolites, and their mutual transformations, may play important roles in apple flower bud formation. The key metabolites in carbon metabolism (such as sucrose, glucose-6-phosphate, and trehalose-6-phosphate) act as a center for regulators that affect plant transition phases and flowering [2,5,6,38]. 

Lipid metabolic substances and their key regulatory genes (such as *FATTY ACID DESATURASE3* and *Wrinkled1*), play important roles in the regulation of floral induction in plants [4,15]. Here, we determined that the proteins involved in lipid transport and metabolism, such as 4-coumarate-CoA ligase 1 and the biotin carboxyl carrier protein of acetyl-CoA carboxylase 2, and acids that are downstream of pyruvate in the fatty acid, lipid, and TCA cycle pathways, such as aconitic, citramalic, glutaric, citric, and shikimic acids, were present at significantly different levels in buds with different flowering rates (Table 1 and Table S1), suggesting that the levels of these metabolites differ among buds with different flowering capabilities in their carbon pathways, including those of sugars, fatty acids, and lipids, and may contribute to apple flower bud formation. Similarly, the phospholipid metabolites that appeared downstream in fatty acid pathways, such as PC, phosphatidylglycerol, and phosphatidylinositol, take part in the regulation of floral induction in plants [15]. Additionally, the proteins encoded by flowering genes can bind to the diurnally changing molecular species of PC in the shoot apex, which mainly involves 18:1-PC, to promote flowering [15]. Furthermore, the greater proportion of 18:3-PC relative to 18:1-PC, produced by overexpression of *FATTY ACID DESATURASE3*, delays flowering [4,15]. 

Proteins are the material bases of life, and amino acids are not only necessary substrates for protein biosynthesis in cells but also participate in the regulation of nitrogen metabolic pathways and the balance between carbon and nitrogen in plants [12,35]. As an important component of plants, the normal physiological metabolism, transport, and transformation of amino acids are necessary for the completion of the life cycle activities of plants [12]. Here, we identified large amounts of metabolites, mainly involved with amino acids and their isoforms, among buds with different flowering capabilities (Table 2 and Table S2), suggesting that they play important regulatory roles in bud growth and flower bud formation. Amino acids, as ‘material bases’ and ‘signal molecules’, play key roles in regulating plant nutrition, growth, and development as well as coping with some abiotic stresses [12,20,21,22,39]. The levels of amino acids and their isoforms (such as putrescine, alanine, proline, threonine, and other polyamines) in the buds with the highest flowering rate (~85%) were significantly greater than those of buds with lower flowering rates (Figure 2 and Figure 10; Table 2, Tables S2 and S3). Similarly, exogenous proline treatments significantly promoted Vigna early flowering [22], and the ectopic expression of the Vigna *P5SC* gene, which encodes an important enzyme for proline synthesis, in tobacco significantly increased the number of flower buds [23]. The overexpression of *AtP5CS1* in *A. thaliana* resulted in early flowering [20], and *AtP5CS2* is an early target gene of *CONSTANS* in flower development [21], suggesting that proline plays an active role in flower bud formation. 

Additionally, polyamines, as important growth regulators of plants, play important roles in cell division and differentiation, embryonic development, fruit setting, flowering, and dormancy [13,14]. However, there is a clear distinction between floral and vegetative buds with respect to the levels of these polyamines, with floral buds containing greater amounts of conjugated polyamines [24]. During flower development, relatively greater levels of polyamines may contribute to the differentiation and maturation processes of flowering in azalea [25]. Indeed, the level of putrescine was significantly greater in Sbs than in the other buds that had lower flowering rates (Table 2; Figure 1). Other polyamines, such as alanine, proline, threonine, phenylalanine, hydroxylamine, and maleimide, showed similar trends (Table 2 and Table S2; Figure 10), suggesting that these amino acid and polyamine metabolites belong to nitrogen pathways and play key roles in floral induction and flower bud formation in apple.

## 4. Materials and Methods

### 4.1. Plant Material and Sample Preparation

Ten-year-old ‘Nagafu No. 2′, a cultivar of ‘Fuji’ apple (*M. domestica* Borkh.) trees grafted on ‘M.26′ rootstocks, were planted in the Apple Demonstration Nursery of Yangling Modern Agriculture Technology Park, Northwest Agriculture and Forestry University, Shaanxi Province, China (34°52′ N, 509 108°7′ E) on 7 March 2008. On 10 October 2017, we collected bud samples with different flowering capabilities from different axillary, long, and spur shoots. These bud samples were frozen immediately in liquid nitrogen and stored at −80 °C until further use. 

### 4.2. Protein Extraction, Digestion, and TMT-Labeling LC–MS/MS

The total proteins of bud samples with different flowering capabilities (Ab, Lb, and Sb) were independently extracted as previously described [40]. Briefly, these bud samples were extracted with Lysis buffer (7 M Urea, 2 M Thiourea, 4% CHAPS, and 40 mM Tris-HCl, pH 8.5) containing 1 mM PMSF and 2 mM EDTA (final concentration). The samples were suspended at 200 W for 15 min and centrifuged at 4 °C, 30,000× *g* for 15 min to produce a supernatant. The supernatant was mixed with 5× volume of chilled acetone for 2 h at −20 °C to precipitate the proteins. After centrifugation at 4 °C, 30,000× *g*, the supernatant was discarded, and the pellet was air-dried for 5 min, dissolved in 500 μL 0.5 M TEAB (Applied Biosystems, Milan, Italy), and sonicated at 200 W for 15 min. Finally, the samples were centrifuged at 4 °C and 30,000× *g* for 15 min. The supernatants were transferred to new tubes and quantified. The proteins in the supernatant were maintained at −80 °C for further analysis. 

The total protein (100 μg) of each bud sample was digested using Trypsin Gold (Promega, Madison, WI, USA) at 37 °C for 16 h. Then, the samples were labeled using TMT tags as follows: sample Abs (126C tag, 127N tag, and 127C tag), Lbs (128N tag, 128C tag, and 129N tag), and Sbs (129C tag, 130N, and 130C). The peptides were labeled with isobaric tags and incubated at room temperature for 2 h. Protein extractions were performed in three biological replicates, with each biological replicate consisting of an independent pool of nine bud samples. The LC-20AB HPLC Pump system (Shimadzu, Kyoto, Japan) was used for identification and fractionation [41]. 

### 4.3. Venn Diagrams and Heatmap Analyses of Differentially Expressed Proteins (DEPs) and Metabolites

Venn diagrams of DEPs and metabolites of buds with different flowering capabilities (Ab, Lb, and Sb) were analyzed [11,31] using VENNTURE software (http://www.irp.nia.nih.gov/branches/lci/nia_bioinformatics_software.html, accessed on 1 July 2021). We used MeV 4.6.2 software to analyze the profiles of DEPs and metabolites (https://www.softpedia.com/get/Science-CAD/MeV.shtml, accessed on 1 July 2021). 

### 4.4. Protein Identification and Function Analyses

We identified the proteins of each bud sample using the Mascot search engine (Matrix Science, London, UK; version 2.3.02) and the apple (*M. domestica* Borkh.) reference genome (https://iris.angers.inra.fr/gddh13, accessed on 20 July 2021). We only used ratios with *p*-values < 0.05, and only fold changes of >1.2 were considered significant. We used a gene ontology (GO) database (http://www.geneontology.org, accessed on 20 July 2021) [42] and the Kyoto Encyclopedia of Genes and Genomes (KEGG) database (http://www.genome.jp/kegg/pathway.html, accessed on 25 July 2021) [43] to analyze the functions of the DEPs, including biological processes, molecular functions, and cellular components, as well as biochemical pathways. A *p*-value < 0.05 was the criterion for significant enrichment. 

### 4.5. Data Processing and Statistical Analyses of Metabolites

We used ChromaTOF software (v4.34, LECO, St Joseph, MI, USA) to analyze the metabolites from GC-MS. Briefly, the resulting data were normalized to the total peak area of each bud sample in Excel 2013 (Microsoft, Washington, DC, USA) and imported into a SIMCA-P software package (version 14.1, Umetrics, Umea, Sweden) where a principal component analysis (PCA), partial least-squares discriminant analysis (PLS-DA), and orthogonal PLS-DA were performed as previously described [44]. The quality of the models was described by the R^2^X or R^2^Y and Q^2^ values. R^2^X or R^2^Y is defined as the proportion of variance in the data explained by the models and indicates goodness of fit. Q^2^ is defined as the proportion of variance in the data predicted by the model and indicates predictability, calculated by a cross-validation procedure. A default seven-round cross-validation in SIMCA was performed throughout to determine the optimal number of PCs and to avoid model overfitting. The orthogonal PLS-DA models were also validated using a permutation analysis [44].

### 4.6. Analysis of Flowering Rates in Buds with Different Flowering Capabilities

We calculated the flowering rates of ‘Nagafu No. 2′ apple buds from different types of shoots, including axillary, long (>10 cm), and spurs (<5 cm). Briefly, we marked 36 shoots of different types from six ‘Nagafu No. 2′ apple trees at the fruit-ripening stage (~20 October) and then counted the numbers of buds on the different shoot types. In the following year, on 10 April 2018, the number of flowering buds was counted. Details of the methods used to calculate flowering rates have been described previously [32,45].

### 4.7. Statistical Analysis

A one-way analysis of variance with Tukey–Kramer multiple comparison tests was performed using DPS software version 7.0 (Zhejiang University, Hangzhou, China). The significance threshold was set at *p* < 0.05 for this test.

## 5. Conclusions

We used a TMT proteomic technique in combination with nLC–MS/MS analyses to compare the proteomic and metabolomic profiles of apple buds with different flowering capabilities. We identified 471 (88 up- and 383 down-regulated), 459 (176 up- and 283 down-regulated), and 548 (387 up- and 161 down-regulated) DEPs, as well as 110 (91 increased and 19 decreased), 89 (71 increased and 18 decreased), and 99 (37 increased and 62 decreased) metabolites that were present at significantly different levels in Sb vs. Lb, Sb vs. Ab, and Lb vs. Ab, respectively. The DEPs were related to carbohydrate synthesis, metabolism and transport processes, amino acid transport and metabolism, and fatty acid and lipid pathways. Additionally, the networks of the metabolites involved in nitrogen metabolism (including amino acids and their isoforms, amines, and polypeptides), carbon metabolism (including polysaccharides, monosaccharides, sugars, and polyols), the TCA cycle, and organic acid metabolism were constructed based on the identified metabolites in buds with different flowering capabilities. Thus, the transitions to flowering and flower bud formation are complex processes that involve various biochemical materials and signals, such as carbohydrates (i.e., sugars and polyols), amino acids and their isoforms, and organic acids.

## Figures and Tables

**Table 1:** Significantly differentially expressed proteins involved in the carbohydrate, amino acid, and lipid metabolism pathways in apple buds with different flowering capabilities.

No.	^a^ Protein ID	Protein Name	Protein Description	Score	^b^ Cov	^c^ Unique Peptides	^d^ MW (kDa)	^e^ Calc. pI	Ratio		
									Sb/Ab	Sb/Lb	Lb/Ab
**Carbohydrate transport and metabolism**											
1	MD10G1063600	FBA2	Probable fructose-bisphosphate aldolase 2	64.9	30.7	3	42.7	7.75	1.03	1.22 *	0.84
3	MD07G1159300	GBSSIb	Granule-bound starch synthase 1	45.4	24.1	9	67.2	7.69	0.82	1.61 *	0.51 *
4	MD07G1226500	F4C21.14	Xyloglucan endotransglucosylase/hydrolase protein 9	100.6	41.3	9	61.7	5.91	0.82	1.37 *	0.6 *
5	MD05G1099400	BXL5	Probable beta-D-xylosidase 5	76.2	22.1	14	87.1	6.87	0.82	1.31 *	0.63 *
6	MD07G1210800	BXL5	Probable beta-D-xylosidase 5	15.9	12.8	1	17.8	7.72	0.99	1.29 *	0.76 *
7	MD02G1049600	RBCL	Ribulose bisphosphate carboxylase large chain	35.2	16.1	6	30.2	5.31	1.15	1.28 *	0.9
8	MD06G1148800	GAPC	Glyceraldehyde-3-phosphate dehydrogenase	100.4	34.5	3	39.8	7.21	1.22 *	1.18	1.04
9	MD09G1073100	PGI	Glucose-6-phosphate isomerase	4.1	4	2	40.7	5.67	0.82	1.14	0.72 *
10	MD12G1028200	INV1	Beta-fructofuranosidase, insoluble isoenzyme 1	30.7	16.3	9	64.6	8.5	1.53 *	1.14	1.34 *
11	MD08G1023600	PpGAL7	Beta-galactosidase 3 (Precursor)	65.7	21.5	9	94.8	7.99	1.32 *	1.13	1.16
12	MD09G1033700	GAPB	Glyceraldehyde-3-phosphate dehydrogenase GAPB	40.9	26.2	3	33.8	8.18	0.9	1.13	0.8
13	MD07G1240700	BXL4	Beta-D-xylosidase 4	16.8	16	5	36.1	5.5	0.51 *	1.11	0.46 *
14	MD06G1002300	PFKa	Pyrophosphate-fructose 6-phosphate 1-phosphotransferase subunit alpha	100.2	39.4	19	67.4	7.21	0.76 *	1.06	0.72 *
15	MD09G1138500	HXK2	Hexokinase-2	117.1	22.5	5	154.1	4.78	1.22 *	0.94	1.29 *
16	MD02G1021200	PAP26	Peroxidase	31.1	21.2	7	55.8	7.78	1.34 *	0.89	1.5 *
17	MD17G1287000	SUS7	Sucrose synthase 7	20.5	6	2	95.8	7.52	1.32 *	0.87	1.52 *
18	MD09G1202200	SUS6	Sucrose synthase 6	25.8	17.9	3	53.6	6.61	1.04	0.86	1.21 *
19	MD09G1088100	HXK1	Hexokinase-1	3.5	1.2	1	84.8	6.57	1.14	0.83	1.38 *
20	MD13G1283100	BGAL9	Beta-galactosidase 9	65	17.6	14	103.5	6.04	1.09	0.82	1.33 *
21	MD09G1214300	SPS1	Probable sucrose-phosphate synthase 1	20.8	7.2	5	106.2	5	1.02	0.76 *	1.35 *
22	MD07G1226000	GBSSIb	Granule-bound starch synthase 1	7.4	4.5	2	67.7	7.78	0.98	0.75 *	1.3 *
23	MD09G1192100	CWINV	Beta-fructofuranosidase, insoluble isoenzyme	10.2	8	5	64.4	8.97	0.86	0.73 *	1.17
**Amino acid transport and metabolism**											
24	MD00G1013000	MDL2	(R)-mandelonitrile lyase 2	59.1	27.5	14	59.6	8.68	0.47 *	0.44 *	1.06
25	MD03G1091100	MDL3	(R)-mandelonitrile lyase 3	63.5	27.6	12	59.7	8.03	0.59 *	0.59 *	0.99
26	MD08G1219100	At4g24830	Argininosuccinate synthase	7.5	6.9	1	53.3	5.97	1.13	0.86	1.3 *
27	MD17G1264900	ASP1	Aspartate aminotransferase	8.1	9.5	2	46.9	7.37	0.69 *	0.77 *	0.9
28	MD10G1254300	CAAT1	Cationic amino acid transporter 1	3.6	1.8	1	66.7	7.2	0.88	0.79 *	1.11
29	MD08G1183900	EMB1144	Chorismate synthase	1.5	2.2	1	35.7	7.94	1.1	0.88	1.25 *
30	MD12G1236000	OAS	Cysteine synthase	60.4	41	1	36	5.6	0.82	0.83	0.99
31	MD08G1207000	GDH1	Glutamate dehydrogenase 1	73.7	30.9	15	71.9	6.99	1.11	0.84	1.32 *
32	MD01G1174400	GDH2	Glutamate dehydrogenase 2	18.1	10.2	1	44.6	6.76	0.92	0.82	1.13
33	MD08G1004100	AATL1	Lysine histidine transporter-like 8	7.8	2.5	1	62.7	7.97	0.79 *	0.75 *	1.05
34	MD15G1141700	MTHFR2	Methylenetetrahydrofolate reductase 2	88.7	39	5	66.5	6.29	0.94	0.81	1.16
35	MD08G1184000	MTHFR2	Methylenetetrahydrofolate reductase 2	19.5	17.5	1	28.8	5.29	1.28 *	0.89	1.45 *
36	MD16G1010700	PTR2	Peptide transporter	14.7	5.3	3	64.7	6.06	0.82	0.77 *	1.06
37	MD11G1081200	PTR5	Peptide transporter	3.3	1.5	1	64.7	8.6	0.86	0.78 *	1.09
38	MD07G1172700	PAL1	Phenylalanine ammonia-lyase 1	63.4	22.1	8	78.5	6.61	0.88	0.71 *	1.24 *
39	MD04G1096200	PAL1	Phenylalanine ammonia-lyase 1	44.1	15.4	5	78.1	6.74	0.69 *	0.7 *	0.99
40	MD00G1037100	DHAPS-1	Phospho-2-dehydro-3-deoxyheptonate aldolase 1	33.9	15.1	4	58.5	8.12	0.7 *	0.72 *	0.98
41	MD15G1372600	DHAPS-1	Phospho-2-dehydro-3-deoxyheptonate aldolase 1	55.7	22.3	1	59.4	8.48	0.83	0.8	1.04
42	MD08G1186700	DHAPS-1	Phospho-2-dehydro-3-deoxyheptonate aldolase 1	53	24.1	3	59.6	8.34	0.9	0.81	1.11
43	MD06G1186500	At5g62680	Probable peptide/nitrate transporter	9.1	6.5	2	48	8.84	0.77 *	0.84	0.91
44	MD05G1102900	SCPL18	Serine carboxypeptidase-like 18	27.2	18.2	4	51.8	5.38	0.89	0.73 *	1.22
45	MD07G1207000	SCPL50	Serine carboxypeptidase-like 50	15.4	5.9	4	75.8	7.2	1.13	0.8	1.4 *
46	MD16G1027500	SCPL50	Serine carboxypeptidase-like 50	24.7	11.2	2	49.4	8.94	0.64 *	0.43 *	1.52 *
**Lipid transport and metabolism**											
47	MD13G1208400	MENB	1,4-Dihydroxy-2-naphthoyl-CoA synthase	4	14.5	1	8.2	5.4	1.03	0.46 *	2.25 *
48	MD02G1229900	CHY1	3-hydroxyisobutyryl-CoA hydrolase 1	17.5	13.2	1	27.1	6.05	0.85	0.77 *	1.11
49	MD17G1229400	4CL1	4-coumarate-CoA ligase 1	28.6	11.5	5	59.5	5.9	0.92	0.76*	1.2 *
50	MD13G1161400	4CL1	4-coumarate-CoA ligase 1	109.6	79.9	5	17.6	6.02	0.98	1.19	0.82
51	MD06G1147500	4CLL9	4-coumarate-CoA ligase-like 9	12.3	7.7	1	60.6	7.43	0.99	0.8	1.24 *
52	MD12G1159600	CAC3	Acetyl-coenzyme A carboxylase carboxyl transferase subunit alpha	102.4	36	11	84.7	8.87	0.79 *	1.05	0.75 *
53	MD08G1051100	CAC3	Acetyl-coenzyme A carboxylase carboxyl transferase subunit alpha	11.5	4.2	2	53	8.34	0.86	0.7 *	1.23 *
54	MD09G1168200	OSCBPY	Beta-amyrin synthase	20.3	12.3	1	87.6	6.93	0.93	0.83	1.12
55	MD17G1159500	OSCBPY	Beta-amyrin synthase	24.4	13.3	2	87.3	6.61	0.78 *	1.05	0.74 *
56	MD13G1274100	OSCBPY	Beta-amyrin synthase	4.8	31.2	2	9.1	5.88	1.02	0.81	1.27 *
57	MD17G1057500	BCCP2	Biotin carboxyl carrier protein of acetyl-CoA carboxylase 2	13.7	6.4	1	29.7	6.89	0.77 *	0.83	0.92
59	MD13G1221200	CASBPX2	Cycloartenol synthase 2	7.6	11	1	10.9	9.7	0.7 *	0.94	0.74 *
60	MD13G1153100	PATL3	Patellin-3	30.2	16.8	4	29.7	10.36	1.26 *	0.72 *	1.74
61	MD10G1282800	DSEL	Phospholipase A1-IIgamma	37.9	24	8	48.6	8.91	0.79 *	1.26 *	0.63 *
62	MD13G1145600	DSEL	Phospholipase A1-IIgamma	4	3.9	1	35.3	7.25	2.71 *	0.74 *	3.67 *
63	MD10G1119700	CXE2	Probable carboxylesterase 2	49.8	61.9	6	10.9	7.24	0.81	1.18	0.69 *
64	MD10G1091100	CXE7	Probable carboxylesterase 7	39	27.7	8	33.4	5.76	1.23 *	0.95	1.3 *
65	MD10G1142100	CXE7	Probable carboxylesterase 7	9.9	5.3	2	78.2	5.16	1.08	0.8	1.35 *
66	MD16G1073700	SBH2	Sphinganine C (4)-monooxygenase 2	5.2	6.6	1	29.5	7.78	0.85	0.77 *	1.09

^a^ Protein ID, according to the Malus domestica Borkh. genome database. ^b^ The proteins that had a statistically significant (*p* < 0.05) Mascot protein score (1.2 or more) from Proteome Discoverer were considered successfully identified. ^c ^COV(95%) indicates the percentage of matching amino acids from identified peptides having a confidence greater than or equal to 95%. ^d^ Unique Peptide, the number of matched unique peptides identified for each protein. ^e^ Ratio, the ratio between intensities of the identified protein among buds with different flowering abilities (e.g., Sb/Lb, Sb/Ab, and Lb/Ab). The ratios that were statistically significant (*p* < 0.05) are indicated with “*”. Ratio changes in the expression level were at least 1.2-fold. The different color content showed numerous changes in the expression levels of the proteins involved in carbohydrate synthesis, metabolism and transport processes, amino acid transport and metabolism, and the fatty acid and lipid pathways. Red indicated significantly up-regulated expression, and green indicated significantly down-regulated expression.

**Table 2:** Identification of the metabolites involved in the sugar, amino acid, and organic acid metabolism pathways that are present at significantly different levels in apple buds with different flowering capabilities.

No.	Peak	Mass	RT (min)	Ratio		
				Sb/Ab	Sb/Lb	Lb/Ab
**Amino acids and their isoforms**						
1	Alanine	116	11.05	1.37 *	1.79 *	0.76 *
2	Proline	142	14.57	1.09	2.02 *	0.54 *
3	Threonine	202	16.03	0.52 *	1.75 *	0.3 *
4	Phenylalanine	120	18.67	0.92	1.39 *	0.66 *
5	Lyxose	160	20.36	1.23 *	2.55 *	0.48 *
6	Tyrosine	100	21.85	1.14	1.28 *	0.89
7	Oxoproline	156	18.26	0.86	1.3 *	0.66 *
8	Isoleucine	158	14.45	0.34 *	1.64 *	0.21 *
9	Serine	204	15.56	1.71 *	2.02 *	0.84
10	Aspartic acid	232	18.13	1.95 *	1.9 *	1.03
11	Valine	144	13.09	0.32 *	1.74 *	0.19 *
12	N-formyl-L-methionine	175	9.33	0.97	1.2 *	0.8
13	N-Acetylisatin	146	10.36	0.92	1.25 *	0.74 *
14	Maleimide	154	10.86	1.52 *	1.37 *	1.11
15	Hydroxylamine	146	11.16	0.95	1.24 *	0.77 *
16	Malonamide	172	18.79	1.79 *	0.27 *	6.58 *
17	Putrescine	174	21.4	1.54 *	1.13	1.36 *
18	N-Methyl-L-glutamic acid	98	13.9	1.94 *	0.49 *	3.94 *
19	Trans-4-hydroxy-L-proline	158	17.38	2.06 *	0.44 *	4.68 *
20	Nicotinoylglycine	208	9.91	0.98	1.18	0.83
21	D-alanyl-D-alanine	188	15.66	0.75 *	4.8 *	0.16 *
22	Nicotinamide	179	24.04	0.53 *	8 *	0.07 *
23	Glutamine	155	11.89	0.57 *	1.09	0.52 *
24	Leucine	158	9.6	0.95	1.18	0.8
**Sugars and polyols**						
25	Sucrose	243	31.8	1.05	1.21 *	0.9
26	Fructose	103	23.29	0.71 *	1.61 *	0.44 *
27	Tagatose	103	23.41	0.75 *	1.49 *	0.5 *
28	Fucose	117	9.27	1.78 *	1.26 *	1.41 *
29	Xylose	160	20.27	1.29 *	2.12 *	0.61 *
30	Cellobiose	231	30.75	0.86	1.44 *	0.6 *
31	Sophorose	204	34.87	1.33 *	1.62 *	0.82
32	Galactose	186	28.12	2 *	2 *	1
33	D-Talose	160	23.64	0.71 *	1.49 *	0.48 *
34	Phytol	143	9.35	1.19	1.77 *	0.67 *
35	Palatinitol	204	34.29	1.32 *	1.76 *	0.75 *
36	Cis-1,2-Dihydronaphthalene-1,2-diol	191	17.07	0.38 *	1.49 *	0.25 *
37	2-Amino-3-methyl-1-butanol	188	11.04	0.91	1.54 *	0.59 *
38	Dodecanol	243	17.02	1.03	1.74 *	0.59 *
39	2-aminoethanethiol	174	10.24	0.93	1.22 *	0.76 *
40	Pyridoxine	281	20.65	0.2 *	2 *	0.1 *
41	D-erythro-sphingosine	204	22.1	1.92 *	1.82 *	1.06
42	Xylitol	129	21.02	1.96 *	1.34 *	1.46 *
43	D-Arabitol	243	21.05	1.31 *	0.81	1.62 *
44	Allo-inositol	191	25.25	1.59 *	0.93	1.71 *
45	Galactinol	204	23.65	1.33 *	1.06	1.25 *
46	Sorbitol	231	21.9	1.91 *	0.79 *	2.42 *
47	Raffinose	306	22.38	0.69 *	0.49 *	1.41 *
48	Myo-inositol	217	26	1.14	1.19	0.96
49	Mannitol	74	24.2	0.81	0.94	0.86
**Organic acids**						
50	Pyruvic acid	174	10.11	1.3 *	1.12	1.16
51	Pipecolinic acid	156	13.16	1.56 *	0.9	1.72 *
52	Aconitic Acid	215	21.35	1.58 *	0.88	1.79 *
53	Galactonic acid	275	23.38	1.66 *	0.9	1.83 *
54	Quinic acid	215	23.14	1.38 *	0.91	1.51 *
55	Citramalic acid	231	17.47	2.06 *	1.17	1.76 *
56	Maleic acid	126	14.6	1.39 *	1.12	1.24 *
57	Gluconic acid	299	21.7	1.58 *	1.58 *	1
58	Nicotinic acid	106	14.52	1.53 *	1.38 *	1.11
59	Benzoic acid	179	13.69	0.98	1.2 *	0.82
60	D-Glyceric acid	189	15.06	1.56 *	1.37 *	1.14
61	Glutaric Acid	192	18.93	1.33 *	1.47 *	0.9
62	Citric acid	183	22.49	1.56 *	1.02	1.52 *
63	Phytanic acid	159	24.42	1.71 *	0.74 *	2.3 *
64	Malonic acid	305	18.22	1.7 *	0.66 *	2.57 *
65	Shikimic acid	204	21.13	1.25 *	0.7 *	1.79 *
66	Capric Acid	117	17.04	2.45 *	0.66 *	3.73 *
67	Saccharic acid	333	25.19	1.27 *	1.14	1.11
68	Cumic Acid	222	9.95	1.01	1.18	0.85
69	Fumaric acid	245	17.49	1.13	2.3 *	0.49 *
70	Succinic acid	147	14.7	0.64 *	0.91	0.7 *
71	Hippuric acid	208	9.56	0.39 *	0.7 *	0.56 *
72	Tartaric acid	204	25.26	0.09 *	0.44 *	0.2 *
73	Threonic acid	220	18.56	0.68 *	0.56 *	1.21 *
74	Lactic acid	117	10.3	0.89	0.73 *	1.22 *

The ratios that were statistically significant (*p* < 0.05) are indicated with “*”. The different color content showed numerous changes in the expression levels of the proteins involved in carbohydrate synthesis, metabolism and transport processes, amino acid transport and metabolism, and the fatty acid and lipid pathways. Red indicated significantly up-regulated expression, and green indicated significantly down-regulated expression.

## Data Availability

The original contributions presented in the study are included in the article/Supplementary Material, further inquiries can be directed to the corresponding author.

## Supplementary Materials

The following supporting information can be downloaded at: https://www.mdpi.com/article/10.3390/plants12233959/s1, Supplementary File S1: Table S1: Significantly differentially expressed proteins involved in Hsp20/alpha crystallin and the cytochrome P450 family in apple buds with different flowering capabilities; Table S2: Identification of metabolites involved in organic acid and other metabolic pathways present at significantly different levels in apple buds with different flowering capabilities; Table S3: Pathways significantly enriched for metabolites in apple buds with different flowering capabilities; Table S4: KEGG pathways of identified metabolites in apple buds with different flowering capabilities; Figure S1: Identification and analysis of proteomes in apple buds with different flowering capabilities. (A) The isoelectric point distribution of identified proteins; (B) the distribution of the peptide count; (C) the molecular mass distribution of identified proteins; and (D) the protein sequence coverage distribution; Figure S2: The correlations of proteomics data in each apple bud sample with different flowering capabilities. Ab: axillary buds with no flowering; Lb: long-shoot buds with a low flowering rate; and Sb: spur buds with a higher flowering rate than the Lb; Figure S3: KEGG pathways enriched with differentially expressed proteins in apple buds with different flowering capabilities. Ab: axillary buds with no flowering; Lb: long-shoot buds with a low flowering rate; and Sb: spur buds with a higher flowering rate than the Lb; Figure S4: Data processing and statistical analyses of metabolites in apple buds with different flowering capabilities. (A–C) Orthogonal partial least-squares discriminant analysis; (D–F) principal component analysis; and (G–I) partial least-squares discriminant analysis. Ab: axillary buds with no flowering; Lb: long-shoot buds with a low flowering rate; and Sb: spur buds with a higher flowering rate than the Lb; Figure S5: The quality of the models described by the R^2^X or R^2^Y and Q^2^ values. Ab: axillary buds with no flowering; Lb: long-shoot buds with a low flowering rate; and Sb: spur buds with a higher flowering rate than the Lb. Supplementary File S2: Additional file S1: The detailed information of identified proteins in buds with different flowering capabilities. Additional file S2: The detailed information of identified metabolites in buds with different flowering capabilities.
