# Supplementary material for: Comparative Proteomic and Metabonomic Profiling of Buds with Different Flowering Capabilities Reveal Novel Regulatory Mechanisms of Flowering in Apple
Source: Plants (Basel). 2023 Nov 24;12(23):3959. doi: 10.3390/plants12233959 (PMC10707870; doi:10.3390/plants12233959)
Supplement: Supplementary file 1 [file plants-12-03959-s001.zip › plants-2595119-supplementary.pdf]

Supporting information

Additional Supporting Information may be found in the online version of this article at the publisher's web-site:

Supplementary File S1

Table S1 Significantly differentially expressed proteins involved in Hsp20/alpha crystallin and the cytochrome P450 family in apple buds with different flowering capabilities.

Table S2 Identification of metabolites involved in organic acids and others metabolic pathways present at significantly different levels in apple buds with different flowering capabilities.

Table S3 Pathways significantly enriched for metabolites in apple buds with different flowering capabilities.

Table S4 KEGG pathways of identified metabolites in apple buds with different flowering capabilities.

Figure S1. Identification and analysis of proteomes in apple buds with different flowering capabilities. (A) The isoelectric point distribution of identified proteins; (B) The distribution of the peptide count; (C) The molecular mass distribution of identified proteins; (D) The protein sequence coverage distribution.

Figure S2. The correlations of proteomics data in each apple bud sample with different flowering capabilities. Ab: axillary buds with no flowering; Lb: long-shoot buds with a low flowering rate; and Sb: spur buds with a higher flowering rate.

Figure S3. KEGG pathways enriched with differentially expressed proteins in apple buds with different flowering capabilities. Ab: axillary buds with no flowering; Lb: long-shoot buds with a low flowering rate; and Sb: spur buds with a higher flowering rate.

Figure S4. Data processing and statistical analyses of metabolites in apple buds with different flowering capabilities. (A–C) Orthogonal partial least-squares discriminant analysis; (D–F) Principal component analysis; (G–I) Partial least-squares discriminant analysis. Ab: axillary buds with no flowering; Lb: long-shoot buds with a low flowering rate; and Sb: spur buds with a higher flowering rate.

Figure S5. The quality of the models described by the R^2X or R^2Y and Q^2 values. Ab: axillary buds with no flowering; Lb: long-shoot buds with a low flowering rate; and Sb: spur buds with a higher flowering rate.

Supplementary File S2

Additional file S1： The detail information of identified proteins in buds with different flowering capabilities.

Additional file S2： The detail information of identified metabolites in buds with different flowering capabilities.

Table S1 Significantly differentially expressed proteins involved in Hsp20/alpha crystallin and the cytochrome P450 family in apple buds with different flowering capabilities.

No.	[a]protein ID	protein Name	Protein Description	[b]Score	[c]Cov	[d]Unique Peptides	[e]MW [kDa]	[f]Calc. pI	[g]Ratio		
									Sb/Ab	Sb/Lb	Lb/Ab
**Hsp20/alpha crystallin family**											
1	MD01G1144400	HPS20	Hsp20/alpha crystallin family	55.8	28.6	3	30.8	9.36	1.25*	2.53*	0.49*
2	MD07G1210700	HPS20	Hsp20/alpha crystallin family	67.9	60	4	17.5	5.49	1.09	1.8*	0.6*
4	MD11G1087100	HPS20	Hsp20/alpha crystallin family	7.9	15.6	3	18.2	6.07	1.21*	1.5*	0.81
5	MD01G1208700	HPS90	Hsp90/alpha crystallin family	91.1	26.7	1	80.7	5.1	1.15	1.25*	0.92
6	MD07G1279100	HPS90	Hsp90/alpha crystallin family	33.3	24.9	1	30.1	9.77	1.18	1.27*	0.93
**Cytochrome P450 family**											
7	MD15G1436500	CYP98A2	Cytochrome P450 98A2	13.6	6.1	1	57.9	8.19	0.68*	0.73*	0.94
8	MD08G1242900	CYP98A2	Cytochrome P450 98A2	25.4	12.5	2	58	8.75	0.8	0.85	0.94
9	MD05G1170000	CYP82C4	Cytochrome P450 82C4	12.3	5.2	1	59.1	7.58	0.66*	0.78*	0.84
10	MD15G1028400	CYP82A4	Cytochrome P450 82A4	15.5	8.7	1	59.3	9.17	0.59*	0.67*	0.88
11	MD08G1234700	CYP82A4	Cytochrome P450 82A4	14.9	13	4	58.9	8.35	0.65*	0.61*	1.07
12	MD15G1028200	CYP82A3	Cytochrome P450 82A3	14.9	8.7	1	58.8	9.07	0.79*	0.75*	1.06
13	MD11G1220400	CYP750A1	Cytochrome P450 750A1	3	1.8	1	57.1	8.25	0.65*	0.62*	1.05
14	MD13G1104100	CYP71B37	Cytochrome P450 71B37	28.6	14.8	3	58.1	7.74	0.83	1	0.83
15	MD13G1103500	CYP71B34	Cytochrome P450 71B34	30.7	12.8	2	57.9	7.2	0.8	0.96	0.84
16	MD06G1163800	CYP71A26	Cytochrome P450 71A26	13.3	7.3	2	60.7	7.91	0.83	0.88	0.94
17	MD11G1219700	CYP71A1	Cytochrome P450 71A1	1.2	1.6	1	56.7	6.84	0.72*	0.65*	1.11

[a]Protein ID, according to the Malus domestica Borkh. genome database.

[c]The proteins that had a statistically significant ($p < 0.05$) Mascot protein score (1.2 or more) from Proteome Discoverer were considered successfully identfied.

[d]COV(95%) indicates the percentage of matching amino acids from identified peptides having confidence greater than or equal to 95%.

[e]Unique Peptide, Number of matched unique peptides identified for each protein.

[f]Ratio, the ratio between intensities of identfied protein among buds with different flowering ability (e.g., Sb/Lb, Sb/Ab and Lb/Ab). The ratios that were statistically signficant ($p < 0.05$) were indicated with “*”. Ratio changes in expression level of at least 1.2-fold.

Table S2 Identification of metabolites involved in organic acids and others metabolic pathways present at significantly different levels in apple buds with different flowering capabilities.

No.	Peak	Mass	RT (min)	Ratio		
				Sb/Ab	Sb/Lb	Lb/Ab
**Organic acids**						
1	2-Furoic Acid	125	11.66	1.24*	1.89*	0.66*
2	3-hydroxybutyric acid	191	11.84	1.11	1.89*	0.59*
3	toluenesulfonic acid	89	15.09	1.11	1.2*	0.92
4	L-Malic acid	147	17.64	1.38*	0.77*	1.79*
5	4-aminobutyric acid	174	18.32	2.56*	2.25*	1.14
6	4-Hydroxybenzoic acid	126	18.81	0.92	1.45*	0.63*
7	3-(4-hydroxyphenyl) propionic acid	179	21.62	0.99	2.25*	0.44*
8	Dehydroascorbic Acid	173	22.96	1.32*	1.23*	1.07
9	Digalacturonic acid	204	28.92	1.22*	1	1.23*
10	beta-Mannosylglycerate	204	19.84	0.91	0.89	1.02
11	Glucose-1-phosphate	217	13.21	1	0.85	1.18
12	3-Aminoisobutyric acid	102	9.78	1.22*	1.48*	0.82
13	caprylic acid	117	13.83	1.53*	1.47*	1.04
14	3,4-dihydroxybenzoic acid	174	22.43	17.7*	0.63*	28.1*
15	D-galacturonic acid	244	25.16	1.41*	2.18*	0.65*
16	Methyl Phosphate	243	12.43	2.36*	1.06	2.23*
17	5-Hydroxyindole-2-carboxylic acid	231	14.4	1.41*	2.28*	0.62*
18	4-hydroxybutyrate	89	16.02	1.62*	0.27*	6*
19	3-Phenyllactic acid	193	21.14	0.52*	4*	0.13*
20	Pelargonic acid	216	15.46	0.92	1.22*	0.75*
**Others**						
1	p-benzoquinone	106	9.28	0.95	1.23*	0.77*
2	2-hydroxypyridine	152	9.97	0.94	1.18	0.8
3	Methoxamedrine	116	11.4	1	1.24*	0.81
4	Aminooxyacetic acid	222	11.74	1.27*	1.47*	0.87
5	1,3-Cyclohexanedione	106	12.17	0.91	1.25*	0.73*
6	Gallic acid	281	12.31	0.97	1.61*	0.6*
7	Dithioerythritol	222	12.53	1.33*	1.43*	0.93
8	uracil	99	15.23	1.16	1.35*	0.86
9	cytidine-monophosphate	243	15.44	0.78*	2.84*	0.28*
10	flavin adenine degrad product	244	20.23	1.32*	2.56*	0.51*
11	ribose	103	20.58	1.09	1.6*	0.68*
12	Digitoxose	186	31.07	1.11	1.91*	0.58*
13	Epicatechin	281	33.73	1.38*	2.42*	0.57*
14	phosphate	299	14.12	2.85*	2*	1.43*
15	Ethanolamine	174	14.01	2.47*	2.17*	1.14
16	Atrazine-2-hydroxy	215	11.83	0.53*	1.68*	0.32*
17	D-erythronolactone	101	15.9	1	0.73*	1.37*
18	Carnitine	117	9.53	0.91	1.24*	0.74*
19	Norleucine	86	12	0.34*	1.51*	0.23*
20	Maleamate	244	10.91	1.43*	0.33*	4.31*
21	21-hydroxypregnenolone	119	10.93	0.8	1.33*	0.6*
22	3-Hydroxypyridine	152	11.77	1.16	1.16	1
23	thymidine	125	13.37	1.08	1.59*	0.68*
24	2-ketoadipate	100	13.55	1.34*	2.53*	0.53*
25	thymine	183	17.41	1	1.67*	0.6*
26	Lyxonic acid, 1,4-lactone	186	21.56	8.67*	0.72*	12*
27	alpha-Ecdysone	171	31.28	2.49*	2.3*	1.08
28	Cellobiotol	204	33.87	1.85*	1.22*	1.53*
29	salicin	179	31.14	1.15	1.9*	0.6*
30	phloretin	179	32.35	1.27*	2.15*	0.59*
31	5-Methoxytryptamine	174	11.01	0.9	1.3*	0.69*
32	2-methylfumarate	185	16.09	1.07	1.67*	0.64*
33	creatine degr	147	10.78	1.03	1.68*	0.61*
34	dibenzofuran	168	11.34	1.2*	1.71*	0.7*
35	Analyte 190	183	15.17	2.93*	1.47*	2*
36	(+)-catechin	179	33.94	2.92*	2.24*	1.31*
37	1-Hydroxyanthraquinone	281	9.39	0.91	1.28*	0.71*
38	3-Cyanoalanine	141	11.29	0.98	1.19	0.82
39	hydroxyurea	222	15.52	1.4*	1.46*	0.96
40	Diglycerol	103	16.4	1.56*	0.87	1.8*
41	5,6-dihydrouracil	171	12.74	0.93	1.6*	0.58*
42	naphthalene	128	21.46	0.87	0.22*	4*
43	5,6-Dimethylbenzimidazole	131	11.49	0.71*	1.61*	0.44*

Table S3 Pathways significantly enriched for metabolites in apple buds with different flowering capabilities.

Pathway Name	Match Status	Raw p	-LOG(p)	Holm adjust	FDR	Impact
Alanine, aspartate and glutamate metabolism	8/22	0.000196	8.5377	0.017047	0.017047	0.44828
Citrate cycle (TCA cycle)	6/20	0.004028	5.5145	0.34642	0.17522	0.25531
Aminoacyl-tRNA biosynthesis	12/67	0.006505	5.0351	0.55295	0.18865	0.09302
Galactose metabolism	6/26	0.015911	4.1407	1	0.34607	0.13132
Arginine and proline metabolism	7/38	0.031259	3.4654	1	0.54391	0.14731
Glyoxylate and dicarboxylate metabolism	4/17	0.044673	3.1084	1	0.64776	0.36054
Valine, leucine and isoleucine biosynthesis	5/26	0.055991	2.8826	1	0.69589	0.03645
Carbon fixation in photosynthetic organisms	4/21	0.087257	2.4389	1	0.94892	0.0331
Pantothenate and CoA biosynthesis	3/14	0.1013	2.2897	1	0.97924	0
Butanoate metabolism	3/18	0.17953	1.7174	1	1	0
Tyrosine metabolism	3/18	0.17953	1.7174	1	1	0.27273
Pyrimidine metabolism	5/38	0.19719	1.6236	1	1	0
Cyanoamino acid metabolism	2/11	0.22739	1.4811	1	1	0
Glycine, serine and threonine metabolism	4/30	0.22911	1.4736	1	1	0.31657
Phenylalanine, tyrosine and tryptophan biosynthesis	3/21	0.2461	1.402	1	1	0.09982
Pyruvate metabolism	3/21	0.2461	1.402	1	1	0.19856
Pentose and glucuronate interconversions	2/12	0.25907	1.3507	1	1	0
beta-Alanine metabolism	2/12	0.25907	1.3507	1	1	0
Nicotinate and nicotinamide metabolism	2/12	0.25907	1.3507	1	1	0
C5-Branched dibasic acid metabolism	1/4	0.29104	1.2343	1	1	0
Glycolysis or Gluconeogenesis	3/25	0.33931	1.0808	1	1	0.10916
Ascorbate and aldarate metabolism	2/15	0.35364	1.0395	1	1	0
Nitrogen metabolism	2/15	0.35364	1.0395	1	1	0
Isoquinoline alkaloid biosynthesis	1/6	0.4033	0.90807	1	1	0.5
Pentose phosphate pathway	2/18	0.44385	0.81228	1	1	0
Starch and sucrose metabolism	3/30	0.45459	0.78836	1	1	0.19711
Tropane, piperidine and pyridine alkaloid biosynthesis	1/8	0.49793	0.69729	1	1	0
Phenylalanine metabolism	1/8	0.49793	0.69729	1	1	0.5
Cysteine and methionine metabolism	3/34	0.54068	0.61492	1	1	0
Valine, leucine and isoleucine degradation	3/34	0.54068	0.61492	1	1	0
Lysine biosynthesis	1/10	0.57767	0.54875	1	1	0
Vitamin B6 metabolism	1/11	0.6127	0.48988	1	1	0
Methane metabolism	1/11	0.6127	0.48988	1	1	0.16667
Sulfur metabolism	1/12	0.64485	0.43874	1	1	0
Glutathione metabolism	2/26	0.64563	0.43752	1	1	0

Glucosinolate biosynthesis	4/54	0.66407	0.40936	1	1	0
Glycerolipid metabolism	1/13	0.67435	0.394	1	1	0
Sphingolipid metabolism	1/13	0.67435	0.394	1	1	0
Propanoate metabolism	1/15	0.72627	0.31983	1	1	0
Fructose and mannose metabolism	1/16	0.74906	0.28893	1	1	0
Lysine degradation	1/17	0.76997	0.2614	1	1	0
Selenoamino acid metabolism	1/19	0.80676	0.21473	1	1	0
Ubiquinone and other terpenoid-quinone biosynthesis	1/23	0.86373	0.14649	1	1	0
Amino sugar and nucleotide sugar metabolism	2/41	0.86598	0.14389	1	1	0.11022
Inositol phosphate metabolism	1/24	0.87515	0.13336	1	1	0.25131
Glycerophospholipid metabolism	1/25	0.88562	0.12147	1	1	0
Terpenoid backbone biosynthesis	1/25	0.88562	0.12147	1	1	0
Phenylpropanoid biosynthesis	2/45	0.89856	0.10696	1	1	0.03823
Tryptophan metabolism	1/27	0.90402	0.1009	1	1	0
Fatty acid biosynthesis	2/49	0.9237	0.079365	1	1	0
Flavonoid biosynthesis	1/43	0.97665	0.023623	1	1	0
Purine metabolism	1/61	0.99535	0.004662	1	1	0

Table S4 KEGG pathways of identified metabolites in apple buds with different flowering capabilities.

Metabolites	Match KEGG NAME	KEGG	HMDB
Norleucine	L-Norleucine	C01933	HMDB0001645
sorbitol	Sorbitol	C00794	HMDB0000247
Isoleucine	L-Isoleucine	C00407	HMDB0000172
valine	L-Valine	C00183	HMDB0000883
Phytanic acid	Phytanic acid	C01607	HMDB0000801
shikimic acid	Shikimic acid	C00493	HMDB0003070
L-Malic acid	L-Malic acid	C00149	HMDB0000156
citric acid	Citric acid	C00158	HMDB0000094
fructose	D-Fructose	C02336	HMDB0000660
succinic acid	Succinic acid	C00042	HMDB0000254
Tagatose	D-Tagatose	C00795	HMDB0003418
fumaric acid	Fumaric acid	C00122	HMDB0000134
quinic acid	Quinic acid	C06746	HMDB0003072
phosphate	Phosphate	C00009	HMDB0001429
Galactonic acid	Galactonic acid	C00880	HMDB0000565
malonic acid	Malonic acid	C00383	HMDB0000691
4-hydroxybutyrate	4-Hydroxybutyric acid	C00989	HMDB0000710
trans-4-hydroxy-L-proline	4-Hydroxyproline	C01157	HMDB0000725
phloretin	Phloretin	C00774	HMDB0003306
D-alanyl-D-alanine	D-Alanyl-D-alanine	C00993	HMDB0003459
Pipecolinic acid	Pipecolic acid	C00408	HMDB0000070
D-Glyceric acid	Glyceric acid	C00258	HMDB0000139
pyridoxine	Pyridoxine	C00314	HMDB0000239
Lyxose	Pectin	C08348	HMDB0003402
threonine	L-Threonine	C00188	HMDB0000167
3-(4-hydroxyphenyl)propionic acid	Desaminotyrosine	C01744	HMDB0002199
proline	L-Proline	C00148	HMDB0000162
Galactinol	Galactinol	C01235	HMDB0005826
oxoproline	Pyroglutamic acid	C01879	HMDB0000267
Maleamate	Maleamate	C01596	METPA0190
2-Furoic Acid	2-Furoic acid	C01546	HMDB0000617
alanine	L-Alanine	C00041	HMDB0000161
phenylalanine	L-Phenylalanine	C00079	HMDB0000159
Phytol	Phytol	C01389	HMDB0002019
Epicatechin	Epicatechin	C09727	HMDB0001871
3,4-dihydroxybenzoic acid	Protocatechuic acid	C00230	HMDB0001856
putrescine	Putrescine	C00134	HMDB0001414
ribose	D-Ribose	C00121	HMDB0000283
Pyruvic acid	Pyruvic acid	C00022	HMDB0000243
sucrose	Sucrose	C00089	HMDB0000258
ferulic acid	trans-Ferulic acid	C01494	HMDB0000954
5-Methoxytryptamine	5-Methoxytryptamine	C05659	HMDB0004095
Gallic acid	Gallic acid	C01424	HMDB0005807
Glucose-1-phosphate	Glucose 1-phosphate	C00103	HMDB0001586

phytosphingosine	Phytosphingosine	C12144	HMDB0004610
cellobiose	Cellobiose	C06422	HMDB0000055
lactic acid	L-Lactic acid	C00186	HMDB0000190
3-Phenyllactic acid	Phenyllactic acid	C01479	HMDB0000779
5,6-dihydrouracil	Dihydrouracil	C00429	HMDB0000076
Dihydroxyacetone	Dihydroxyacetone	C00184	HMDB0001882
21-hydroxypregnenolone	21-Hydroxypregnenolone	C05485	HMDB0004026
Pelargonic acid	Pelargonic acid	C01601	HMDB0000847
5,6-Dimethylbenzimidazole	Dimethylbenzimidazole	C03114	HMDB0003701
Capric Acid	Capric acid	C01571	HMDB0000511
3-Aminoisobutyric acid	3-Aminoisobutanoic acid	C05145	HMDB0003911
Hippuric acid	Hippuric acid	C01586	HMDB0000714
Dodecanol	Dodecanol	C02277	HMDB0011626
Carnitine	L-Carnitine	C00318	HMDB0000062
2-aminoethanethiol	Cysteamine	C01678	HMDB0002991
mannitol	Mannitol	C00392	HMDB0000765
dibenzofuran	Dibenzofuran	C07729	METPA0948
benzoic acid	Benzoic acid	C00180	HMDB0001870
glutamine	L-Glutamine	C00064	HMDB0000641
D-Arabitol	D-Arabitol	C01904	HMDB0000568
p-benzoquinone	Quinone	C00472	HMDB0003364
4-Hydroxybenzoic acid	4-Hydroxybenzoic acid	C00156	HMDB0000500
D-galacturonic acid	Galacturonic acid	C08348	HMDB0002545
2-hydroxypyridine	2-Hydroxypyridine	C02502	HMDB0013751
serine	L-Serine	C00065	HMDB0000187
Aconitic Acid	cis-Aconitic acid	C00417	HMDB0000072
uracil	Uracil	C00106	HMDB0000300
xylose	D-Xylose	C00181	HMDB0000098
Ethanolamine	Ethanolamine	C00189	HMDB0000149
gluconic acid	Gluconic acid	C00257	HMDB0000625
nicotinic acid	Nicotinic acid	C00253	HMDB0001488
myo-inositol	myo-Inositol	C00137	HMDB0000211
4-aminobutyric acid	Gamma-Aminobutyric acid	C00334	HMDB0000112
aspartic acid	L-Aspartic acid	C00049	HMDB0000191
galactose	D-Galactose	C00984	HMDB0000143
hydroxyurea	Hydroxyurea	C07044	HMDB0015140
4-Hydroxycyclohexanecarboxylic acid	4-Hydroxycyclohexylcarboxylic acid		HMDB0001988
Dehydroascorbic Acid	Dehydroascorbic acid	C00425	HMDB0001264
maleic acid	Maleic acid	C01384	HMDB0000176
Saccharic acid	Glucaric acid	C00818	HMDB0000663
(+)-catechin	Epicatechin	C09727	HMDB0001871
asparagine	L-Asparagine	C00152	HMDB0000168
Glutaric Acid	Glutaric acid	C00489	HMDB0000661
xylitol	D-Xylitol	C00379	HMDB0002917
Citramalic acid	Citramalic acid	C00815	HMDB0000426
fucose	L-Fucose	C01019	HMDB0000174
caprylic acid	Caprylic acid	C06423	HMDB0000482
2-ketoadipate	Oxoadipic acid	C00322	HMDB0000225

hydroxylamine	Hydroxylamine	C00192	HMDB0003338
N-Acetylisatin	N-Acetylisatin	C02172	METPA0257
tyrosine	L-Tyrosine	C00082	HMDB0000158
N-formyl-L-methionine	N-Formyl-L-methionine	C03145	HMDB0001015
Cumic Acid	4-Isopropylbenzoic acid	C06578	HMDB0035268
Nicotinoylglycine	Nicotinuric acid	C05380	HMDB0003269
2-methylfumarate	Mesaconic acid	C01732	HMDB0000749
Threonic acid	Threonic acid	C01620	HMDB0000943
thymidine	Thymidine	C00214	HMDB0000273
3-hydroxybutyric acid	3-Hydroxybutyric acid	C01089	HMDB0000357
raffinose	Raffinose	C00492	HMDB0003213
thymine	Thymine	C00178	HMDB0000262
salicin	Salicin	C01451	HMDB0003546
leucine	L-Leucine	C00123	HMDB0000687

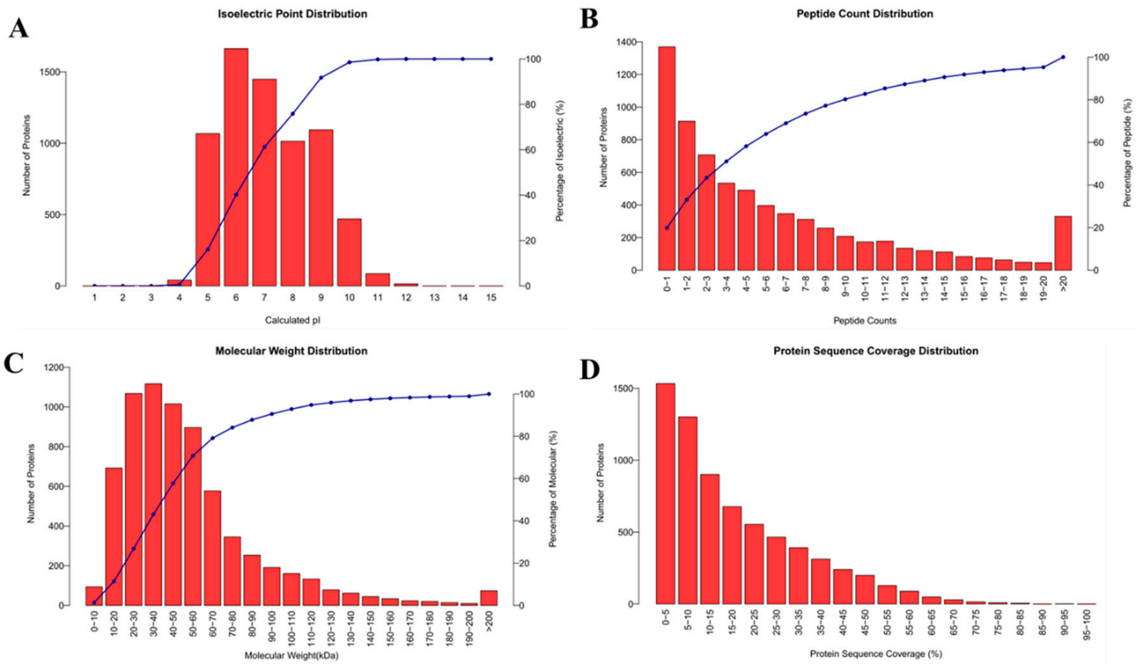

Figure S1. Identification and analysis of proteomes in apple buds with different flowering capabilities. (A) The isoelectric point distribution of identified proteins; (B) The distribution of the peptide count; (C) The molecular mass distribution of identified proteins; (D) The protein sequence coverage distribution.

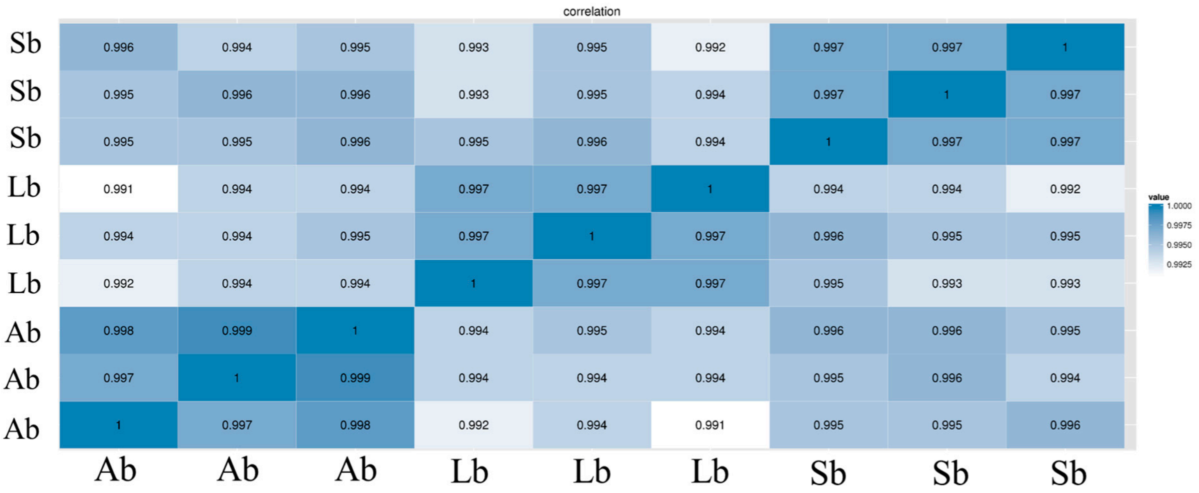

Figure S2. The correlations of proteomics data in each apple bud sample with different flowering capabilities. Ab: axillary buds with no flowering; Lb: long-shoot buds with a low flowering rate; and Sb: spur buds with a higher flowering rate.

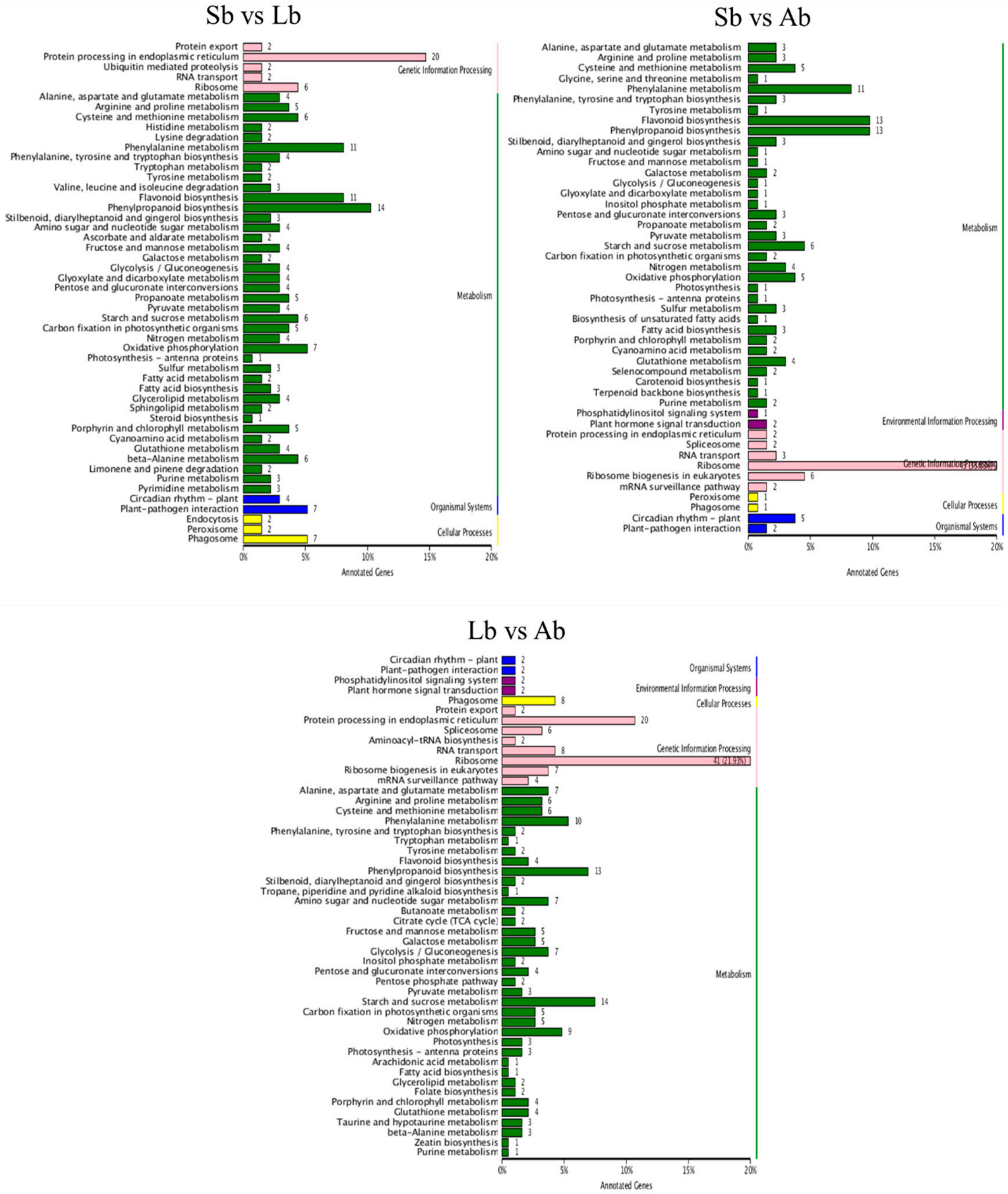

Figure S3. KEGG pathways enriched with differentially expressed proteins in apple buds with different flowering capabilities. Ab: axillary buds with no flowering; Lb: long-shoot buds with a low flowering rate; and Sb: spur buds with a higher flowering rate.

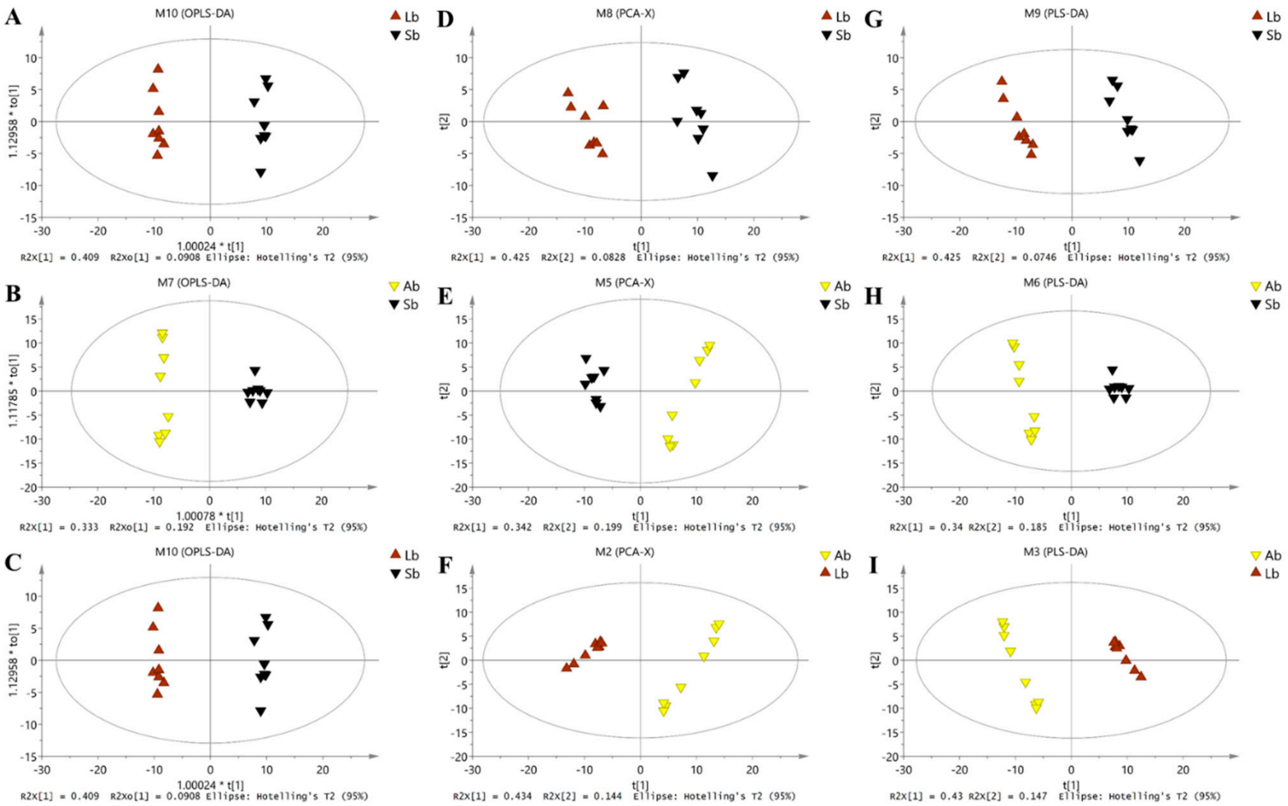

Figure S4. Data processing and statistical analyses of metabolites in apple buds with different flowering capabilities. (A–C) Orthogonal partial least-squares discriminant analysis; (D–F) Principal component analysis; (G–I) Partial least-squares discriminant analysis. Ab: axillary buds with no flowering; Lb: long-shoot buds with a low flowering rate; and Sb: spur buds with a higher flowering rate.

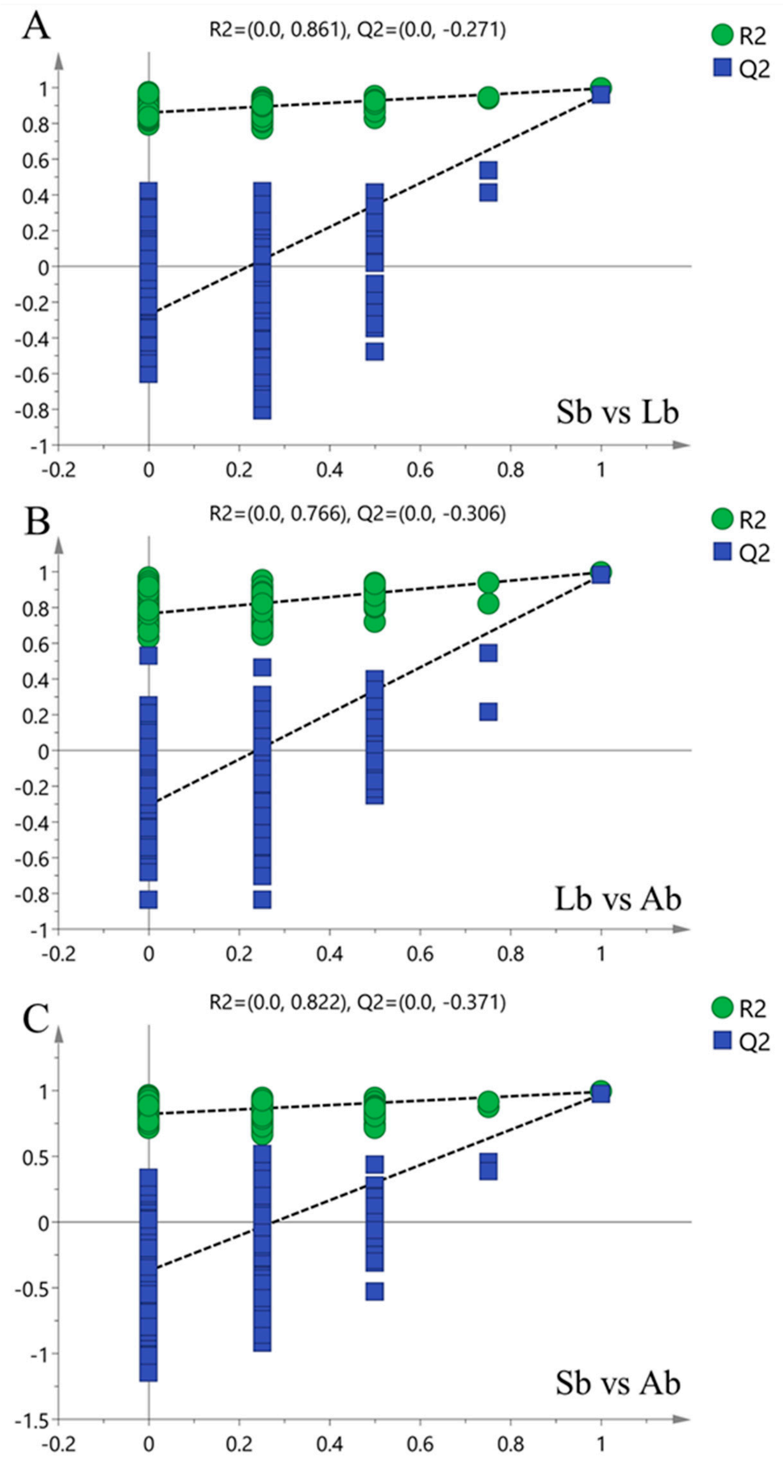

Figure S5. The quality of the models described by the R^2X or R^2Y and Q^2 values. Ab: axillary buds with no flowering; Lb: long-shoot buds with a low flowering rate; and Sb: spur buds with a higher flowering rate.
